# Targeting Cancer-Associated PCNA with AOH1996 Induces Mitotic Catastrophe and Enhances Cisplatin Therapy in Cervical Cancer

**DOI:** 10.1158/2767-9764.CRC-25-0648

**Published:** 2026-05-27

**Authors:** Sebastian O. Wendel, Grant M. Brooke, Changkun Hu, Allison R. Sandoval, Pouya Haratipour, Long Gu, Malaney Young, Maryam Zangi, Brittany L. Rasche, N.S. Banerjee, Jennifer Jossart, Kelly Garvin, Brian V. Geisbrecht, Rachel Cianciolo, Jacob Cawley, J. Jefferson P. Perry, Robert J. Hickey, Linda H. Malkas, Nicholas A. Wallace

**Affiliations:** 1 College of Health and Human Science, Kansas State University, Manhattan, Kansas.; 2 Division of Biology, Kansas State University, Manhattan, Kansas.; 3 Basic Sciences Division, Howard Hughes Medical Institute, Fred Hutchinson Cancer Center, Seattle, Washington.; 4 Department of Cancer Biology and Molecular Medicine, Beckman Research Institute of City of Hope, Duarte, California.; 5 Department of Molecular Diagnostics and Experimental Therapeutics, Beckman Research Institute of City of Hope, Duarte, California.; 6 College of Veterinary Medicine, Kansas State University, Manhattan, Kansas.; 7 Department of Biochemistry and Molecular Genetics, Heersink School of Medicine, Birmingham, Alabama.; 8 Department of Clinical Sciences, Colorado State University, Fort Collins, Colorado.; 9 Department of Biochemistry and Molecular Biophysics, Kansas State University, Manhattan, Kansas.; 10 Department of Veterinary Biosciences, College of Veterinary Medicine, Ohio State University, Columbus, Ohio.; 11 HHS Deans Office, Kansas State University, Manhattan, Kansas.

## Abstract

Cervical cancers remain a significant health burden. Limitations on cervical cancer chemotherapeutic intervention caused by toxic side effects are a persistent barrier to care. In this study, we show that the human papillomavirus oncogenes that cause most cervical cancers also increase the levels of a cancer-associated isoform of proliferating cell nuclear antigen (PCNA) known as caPCNA. The abundance of caPCNA is specifically elevated in cervical cancer. Similar to observations in other cancers, we found that a small-molecule inhibitor of caPCNA (AOH1996) selectively killed cell line, organoid, and xenograft models of cervical cancer. Our subsequent molecular analysis identified a novel ability of AOH1996 to induce cell death by disrupting the interaction between PCNA and γ-tubulin, resulting in mitotic arrest. We show AOH1996 selectively induces mitotic death in transformed cells, because these cells attempt to progress through mitosis, rather than decondensing their chromosomes and reforming their nuclear membranes like untransformed control cells. Furthermore, we show that these differences allow AOH1996 to specifically sensitize cervical cancer cells to cisplatin, a frontline chemotherapeutic used to treat cervical cancer. We found that subtherapeutic doses of AOH1996 and cisplatin could reduce cervical cancer xenograft growth and improve survival, similarly to a therapeutic dose of cisplatin without the cisplatin-induced toxicity that restricts care. To our knowledge, this study provides the first evidence that AOH1996 can function as a cisplatin-sensitizing agent in cervical cancer models.

**Significance:**

We identify a novel mechanism by which the small-molecule inhibitor AOH1996 targets cancer-associated PCNA to induce mitotic death in cervical cancer cells. By disrupting PCNA:γ-tubulin interactions, AOH1996 selectively sensitizes tumors to a lower dose of cisplatin, enabling effective therapy with reduced toxicity and suggesting a potential strategy to reduce treatment-associated toxicity.

## Introduction

Cervical cancer is the fourth most common malignancy among women, accounting for more than 300,000 deaths annually (1). Most cervical cancers are caused by persistent infection with high-risk human papillomaviruses (HPV), particularly HPV16 and HPV18, which drive oncogenesis and tumor maintenance through continual HPV oncogene (E6 and E7) expression (2, 3). Although HPV vaccination and screening programs can reduce cervical cancer, infrastructural barriers limit the efficacy of these approaches in the developing world, where most cervical cancers occur (4). As a result, many patients are diagnosed at advanced stages, in which standard treatments are far less effective (5). As preventive measures do not offer equal protection to all, cervical cancer will remain a substantial global health challenge, particularly in settings with limited access to screening and vaccination.

As a result, there is an ongoing need to improve cervical cancer care. There are at least two major barriers to accomplishing this goal. First, current first-line therapies for advanced cervical cancer (cisplatin-based chemotherapy and radiotherapy) have harsh, often dose-limiting toxicities, including nephrotoxicity, neurotoxicity, and ototoxicity (6–9). Second, there is no widely accepted second-line therapy for tumors that have innate or acquired resistance to cisplatin (10). There is a need for novel approaches that remain effective against cisplatin-resistant cells and for sensitizing agents that make existing therapeutics effective at lower, less toxic doses.

One way to overcome these challenges is to develop targeted therapies that exploit alterations in gene expression in cervical cancer. Because most cervical cancers require constitutive HPV oncogene expression, expression changes caused by HPV oncogenes are often maintained in tumors. Thus, characterizing HPV oncogene biology is an important way to predict cervical cancer biology. We have shown characterizing changes in cell signaling resulting from expressing HPV oncogenes in primary cell lines can lead to clinically relevant insights, including mechanisms of acquired and innate cisplatin resistance/sensitivity (11–13). Furthermore, many of the expression changes identified using these cell culture models are later found to be phenocopied in HPV-negative (HPV−) tumors (e.g., p53 inactivation; ref. 14).

Another relevant HPV oncogene induced change in cell signaling is the increase in the abundance of proliferating cell nuclear antigen (PCNA), a DNA clamp essential for replication and repair. These changes have recently been leveraged to serve as a potential biomarker for cervical cancer (15). The elevated expression of PCNA is also an attractive target for therapeutic inhibition, as PCNA serves as a critical hub for DNA metabolism, DNA repair, cisplatin resistance, proliferation, and centrosome activity (16–19). However, because PCNA is an essential gene in transformed and untransformed cells, it is difficult to inhibit without systemic toxicity (17). One approach to overcoming this challenge has been to gain specificity by targeting specific PCNA:protein interactions (20, 21). However, these compounds have not been successfully developed for use in humans.

To increase the cancer specificity of PCNA inhibitors, we have characterized a cancer-associated isoform of PCNA (caPCNA), distinguished from normal PCNA by a shift in the isoelectric point stemming from changes at the interdomain connecting loop (IDCL), a region involved in binding partner recognition (22). This change is rare in normal cells but prevalent in malignant cells. We have also developed a small-molecule inhibitor, AOH1996, that binds the modified IDCL region and disrupts caPCNA-mediated protein interactions. AOH1996 demonstrates selective cytotoxicity in pancreatic, neuroblastoma, small cell lung cancer, leukemia, and breast cancer models, both *in vitro* and *in vivo*, with no apparent toxicity to untransformed tissues (23–26). AOH1996 can kill cancer cells by enhancing the interaction between caPCNA and the largest subunit of RNA polymerase II (RPB1), leading to cancer-specific impaired transcription replication conflict resolution, RPB1 degradation, and replication fork collapse in actively transcribed chromatin (24). This mechanism drives DNA damage and cancer cell death *in vitro* and *in vivo*, without affecting untransformed cells. However, the PCNA interactome is expansive, and as a result AOH1996 likely has additional mechanisms of action.

Here, we show that AOH1996 selectively kills a broad panel of cervical cancer cell lines, as well as organoid and xenograft models of cervical cancer. This selective toxicity can be driven by HPV oncogenes but also arises in HPV− cervical cancer. We also identified a novel mechanism of action for AOH1996, showing that the inhibitor interferes with the interaction between PCNA and γ-tubulin, disrupting centrosome and spindle organization, arresting cells in mitosis and causing mitotic death. We generated a novel analog of AOH1996, gaining insight into the structurally important aspects of the inhibitor. Furthermore, we have shown that these properties of AOH1996 synergize with cisplatin *in vitro* and *in vivo*. Our cervical cancer xenograft studies demonstrate that this sensitization allows cisplatin to effectively manage tumor growth and improve survival while reducing treatment-associated toxicities. These findings expand the clinical utility of AOH1996 by providing a strong argument for AOH1996-based combination therapy.

## Materials and Methods

### 2-Dimensional gel electrophoresis

Mock-transduced HFK cells were grown and harvested at passage 6. HFK cells transduced with HPV 16 E6E7 were grown and harvested at passages 22 and 62. The cell pellets were then lysed by incubating in a homogenization buffer (50 mmol/L Tris, 250 mmol/L sucrose, 5 mmol/L EDTA, 5 mmol/L EGTA, 1 mmol/L DTT, and 1× Halt protease inhibitor) and then subjected to 50 strokes with a loose-fitting Dounce homogenizer on ice. The homogenate was incubated on ice for 20 minutes prior to centrifugation at 1,000 *g* for 15 minutes to harvest the nuclear pellet. The nuclear pellet was resuspended in a detergent soluble buffer (10 mmol/L Tris, 1 mmol/L magnesium chloride, 1% NP-40, 1 mmol/L DTT, 0.5 U/μL Benzonase, and 1× Halt protease inhibitor), incubated on ice for 10 minutes, sonicated at 30% amplitude, rotated at 4°C for 30 minutes, and pelleted at 18,000 *g* for 10 minutes to harvest the nuclear lysate. The protein concentration of the nuclear lysate was then determined using Pierce 660 nm assay.

The nuclear lysate was desalted using a Zeba desalting column immediately prior to running the first dimension. Isoelectric focusing tube gels containing 3/10 ampholytes were assembled onto a gel electrophoresis cell. Thirty μg of nuclear lysate from both the mock-transduced and transduced HFK cells were loaded onto the tube gels and ran at 500 V for 10 minutes followed by 750 V for 3.5 hours. The second dimension was run by placing the tube gels onto a 12% SDS-PAGE gel and subjected to electrophoresis at 180 V for 30 minutes. The gels were then transferred to a polyvinylidene difluoride membrane using standard Western blotting technique, and the membranes were probed for PCNA using a PC10 (Santa Cruz, RRID: AB_628110) antibody.

### Synthesis of AOH1996 and AOH1996-8Nq

AOH1996 was synthesized using our previously described procedure (24), and AOH1996-8Nq was synthesized as described here. Purification of designated intermediates and final compounds was performed using an ISCO CombiFlash chromatography system equipped with UV detector. All of the other reagents were purchased from Ambeed, Sigma-Aldrich, TCI, or Alfa Aesar (reagent grade) and used as received. ^1^H and ^13^C NMR spectra were obtained using a 700 MHz Bruker Avance spectrometer. All of the ^1^H and ^13^C peak assignments for the final compounds were verified by COSY and HSQCAD. Mass spectrometry (MS) was performed on an Advion CMS equipped with ESI ion source in both positive and negative modes. MS m/z values were calculated using ChemDraw 20.1.1.125 (RRID: SCR_016768). Compound IUPAC names were assigned using ChemDraw 20.1.1.125. The molar yields of the final products were calculated by weighing dry compounds.

Synthesis of *N*-(2-((2-(3-methoxyphenoxy)phenyl)amino)-2-oxoethyl)quinoline-8-carboxamide (AOH1996-8Nq): According to our previously described procedure (24), 90 mg (0.52 mmol, 1.1 equiv.) of quinoline-8-carboxylic acid (purchased from Ambeed) in anhydrous DCM/THF/DMF (10:3:1; 0.2 mol/L) was added using DIPEA (3.3 equiv.) and activated by 197 mg HBTU (0.52 mmol, 1.1 equiv.) for 10 minutes. Then, 128 mg (0.47 mmol, 1 equiv.) of compound 2-amino-*N*-(2-(3-methoxyphenoxy)phenyl)acetamide (24) was added to the solution mixture and stirred under a nitrogen atmosphere for 18 hours. After completion of the reaction (monitored by TLC), the volatile reaction components were removed under reduced pressure. The crude product was redissolved in 50 mL DCM and washed with 25 mL of saturated Na_2_CO_3_ solution and 25 mL of brine solution and then purified by Combi*Flash* chromatography, with a gradient wash (ethyl acetate/hexane) to produce 202 mg (MW: 427.4 g/mol, 0.47 mmol, quantitative yield) title compound. ^1^H NMR (700 MHz, CDCl_3_) δ 11.85 (t, *J* = 6 Hz, 1H), 8.92 (s, 1H), 8.82 (dd, *J* = 4.3, 1.8 Hz, 1H), 8.70 (dd, *J* = 7.3, 1.6 Hz, 1H), 8.46 (dd, *J* = 8.3, 1.6 Hz, 1H), 8.25 (dd, *J* = 8.3, 1.8 Hz, 1H), 7.95 (dd, *J* = 8.1, 1.6 Hz, 1H), 7.61 (t, *J* = 7.7 Hz, 1H), 7.44 (dd, *J* = 8.3, 4.2 Hz, 1H), 7.12 (ddd, *J* = 8.4, 7.5, 1.4 Hz, 1H), 6.99 (td, *J* = 7.6, 1.6 Hz, 1H), 6.90 to 6.81 (m, 2H), 6.34 (dt, *J* = 8.1, 2 Hz, 1H), 6.23 (dt, *J* = 7, 2 Hz, 2H), 4.41 (d, *J* = 5.8 Hz, 2H), 3.57 (s, 3H). ^13^C NMR (176 MHz, CDCl_3_) δ 168.50, 167.07, 160.85, 158.17, 149.69, 145.56, 145.05, 138.03, 134.40, 132.65, 130.60, 130.04, 128.67, 127.75, 126.71, 124.89, 124.46, 121.29, 121.26, 119.48, 109.49, 108.94, 103.45, 55.39, 45.60. MS (ESI+) m/z: (M + H)^+^ calcd for C_25_H_22_N_3_O_4_^+^ 428.1; found 428.2.

### Surface plasmon resonance

Surface plasmon resonance (SPR) was performed by immobilizing porcine tubulin (BK006P, Cytoskeleton Inc.) on an HC1500 SensorChip (Xantec Bioanalytics GmbH), according to the general methods described previously (27). Flow cell 1 was used as a reference surface, whereas flow cells 2, 3, and 4 were modified with 23,715, 18,230, and 15,168 RU of porcine tubulin, respectively. HBST [20 mmol/L HEPES (pH 7.4), 140 mmol/L NaCl, and 0.005% (v/v) Tween-20] supplemented with 5% (v/v) DMSO was used as the running buffer. Concentrated stock solutions of AOH1996 or AOH1996-8Nq were prepared initially in neat DMSO; this working stock was diluted 20-fold with 1.05×-HBST, and thereafter serial twofold dilutions (400–6.25 μmol/L) were prepared in running buffer. Samples were injected over the sensor chip using a reference subtraction mode, while including a solvent-correction curve at the beginning of the experiment and after each 50 cycles. Each injection included a 30-second association phase followed by a 30-second dissociation phase. Surface regeneration was achieved using a 60-second pulse of 20 mmol/L glycine (pH 2.2) and 1.5 mol/L NaCl. Reference-subtracted sensorgrams were processed using Biacore T200 Evaluation Software (v3.2.1, Cytiva LifeSciences). Sensorgrams were analyzed by plotting the response versus analyte concentration and fitting to a dose–response curve to derive apparent K_D_ estimates and associated errors from replicate observations (*n* = 9) using GraphPad Prism v10.3 (RRID: SCR_002798).

### PCNA thermal shift assay

Recombinantly expressed PCNA protein, AOH1996-8Nq, and 200× SYPRO orange dye (Sigma) were diluted into Dulbecco’s phosphate buffered saline (PBS) solution (Sigma). The final concentration of PCNA was 3 μmol/L, the final compound concentrations were at 0, 0.781, 1.56, and 3.13 μmol/L, and the assay was performed in triplicate. Using a Bio-Rad CFX Connect Real-Time PCR Detection System, sample plates were heated from 25°C to 95°C with heating increments of 0.5°C/minute. Fluorescence intensity was measured within the excitation/emission ranges 470 to 505/540 to 700 nm.

### Tissue culture

The C33a (ATCC HTB-31, RRID: CVCL_1094) cell line was purchased from the ATCC. CaSki cells were a generous donation from the Katzenellenbogen laboratory. HeLa (RRID: CVCL_0030) and HeLa POLη cells were generously donated by the Cohn laboratory and thoroughly characterized previously by us and other laboratories (11, 28). Control human foreskin keratinocytes (HFK) were obtained in-house from anonymous medical waste. Cell lines were either grown (37C, 5% CO_2_, humidified) in DMEM (Corning, 10-013-CV), supplemented with 10% FBS (VDR Life Sciences, 76308-942; CaSki, C33a, HeLa, HeLa POLη), or maintained in Keratinocyte Growth Medium 2 (PromoCell) supplemented with the Supplement Mix from PromoCell (HFKs, CaSki, C33a, HeLa, and HeLa POLη). Cervical cancer cell lines were authenticated via short tandem repeat profiling by the ATCC. All cell lines were checked for *Mycoplasma* monthly. Cervical cancer cell lines were obtained from colleagues (Denise Galloway and Rachel Katzenellenbogan). Their passage numbers are undetermined as is the case with most widely used and long-established transformed cell lines. Oncogene expressing HFKs were generated in our lab using retroviral systems to express these HPV genes at physiologically relevant levels. Their expression was confirmed by decreases in established degradation targets. HFKs were passaged between 5 and 10 times.

### MTT assays

Cells were seeded in 96-well plates (CELLTREAT) at a density of 4,000 cells per well. After letting cells attach overnight, cells were treated with AOH1996 (0, 0.025, 0.05, 0.1, 0.25, 0.5, 1, 1.5, and 2 μmol/L). Cells were treated for 72 hours, after which the medium was removed and replaced with fresh medium containing 10% (v/v) MTT (5 mg/mL in PBS) reagent (Thermo Fisher Scientific, M6494). After 24 hours, 100 μL solubilization solution (10% SDS in 0.01 mol/L HCl) was added, and absorption was measured at 550 nm and corrected with the absorption at 690 nm (BioTek Synergy LX plate reader).

For MTT experiments measuring synergy between AOH1996 and cisplatin, cells were seeded as described above. The plate was then pretreated with AOH1996 (0, 0.1, 0.2, 0.3, 0.4, 0.5, 1, and 2 μmol/L) vertically increasing doses. AOH1996 medium was removed and replaced with cisplatin medium (0, 0.5, 1, 2, 3, and 5 μmol/L) in horizontally ascending doses. This generates a dose-matrix with a unique treatment for each well. Plates were then incubated for 72 hours and subsequently analyzed as described above. To detect synergy, we used synergy finder 3.0 as described here (29).

### Annexin V/propidium iodide apoptosis assay

Cells were grown in six-well plates at 250,000 cells per well. After overnight attachment, the cells were treated with 1 μmol/L AOH1996 for 48 hours. This dose was chosen to ensure that the compound does not elicit cell death in primary cells, even at higher doses. Cells were then lifted and stained according to the Annexin V/propidium iodide (PI) manufacturer’s protocol (BioLegend). The live-cell population was analyzed using an Accuri C6 Plus flow cytometer (BD Bioscience) with 533/30 (AnnexinV) and 585/40 (PI) filters. Cells were gated to exclude debris via SSC/FSC and split into quadrants according to their Annexin V and PI signals. Data were analyzed using C6 Plus software and GraphPad Prism.

### Organoid cultures

Organoid cultures of the cervical cancer cell line, CaSki, was prepared as described before (30). Briefly, dermal equivalents (DE) were prepared by mixing rat tail collagen with J2 fibroblasts in 24-well plates. After congelation, the DEs were cultured in organoid raft culture media for 6 to 12 hours and then overlaid with CaSki cells from 80% confluent plates. After another 12 to 24 hours, the assembly was raised to air–media interface and cultured for 7 to 10 days, when the treatments were started. The AOH1996 doses were chosen to account for transport resistance through the collagen–fibroblast plug. Media, with or without inhibitors, were refreshed every 48 hours. At the end of treatment, the CaSki origin recognition complexs were harvested, fixed in 10% buffered formalin, and paraffin-embedded for staining. Stained sections were analyzed by a blinded, board-certified pathologist.

### Cell-cycle profile

Cells were grown in six-well plates at 250,000 cells per well. After overnight attachment, cells were treated with 1 μmol/L AOH1996 for 24 or 48 hours. The cells were then lifted, fixed for 30 minutes with 70% cold ethanol, washed with PBS, treated with 50 μL of a 100 μg/mL stock solution of RNase A (New England Biolabs), and stained with 200 μL of 50 μg/mL PI. The fixed cells were then analyzed using an Accuri C6 Plus flow cytometer (BD Biosciences). Debris was excluded via SSC/FSC gating, and doublets were excluded by the FSC height over the FSC area.

### Western blot

After 24 hours of treatment with 1 μmol/L AOH1996, lysates were generated and immunoblots were run as previously described (31). The membranes were probed with the following antibodies: 1:200 phosphorylated histone H3 (p-H3) Ser10 (Cell Signaling Technology, 9701S, RRID: AB_331535), 1:1,000 GAPDH (Santa Cruz Biotechnology, sc-47724, RRID: AB_627678), 1:1,000 pericentrin (Abcam, ab4448, RRID: AB_304461), 1:2,000 ATAD5 (Novus, NB100-57495, RRID: AB_2060745), 1:1,000 γ-tubulin (Sigma-Aldrich, T5326, RRID: AB_532292), 1:1,000 β-tubulin (Cell Signaling Technology, 86298S, RRID: AB_2715541), and 1:1,000 nucleolin (Santa Cruz Biotechnology, sc-8031, RRID: AB_670271). After incubation with the corresponding horseradish peroxidase (HRP)-conjugated secondary antibody, the blots were visualized using the SuperSignal West Femto maximum sensitivity substrate (Thermo Fisher Scientific).

### Mitotic spreads

HFK, HeLa, or HeLa POLη cells were grown on 10 cm^2^ plates to 40% confluency and treated with 1 μmol/L AOH1996 for 24 hours. Cells were dropped onto microscopy slides and imaged via DIC microscopy, as previously described (32).

### Immunofluorescent microscopy

Cells were seeded in six-well plates at a concentration of 100,000 cells/well in 2 mL of medium. After attachment, the cells were treated with 200 nmol/L AOH1996 for 24 or 48 hours. Due to the large amount of detached mitotic cells, samples were spun onto microscopy slides at 1,000 rpm for 5 minutes via cytospin (Thermo Fisher Scientific). The cells were fixed and stained as previously described (11). Primary antibodies, γ-tubulin 1:500 (Sigma-Aldrich, T5192, RRID: AB_261690), 1:100 β-tubulin (Cell Signaling Technology, 86298S, RRID: AB_2715541), and 1:200 p-H3 Ser10 (Cell Signaling Technology, 9701S, RRID: AB_331535) were used to incubate overnight and then stained with corresponding secondary antibodies AF-488 and AF-594, 1:500 (Invitrogen, A11001, RRID: AB_2534069 and A11012, RRID: AB_2534079, respectively). Images were analyzed using a previously established protocol using FIJI (RRID: SCR_002285) and Zeiss Zen Blue software (RRID: SCR_013672; ref. 33).

### Live-cell microscopy

HFK-Tert, HeLa, and HeLa POLη cells were transduced with a commercial lentiviral vector (VectorBuilder) of LaminB1-GFP/H2B-RFP according to the manufacturer’s protocol. This allows visualization of the nucleus (LaminB1) and DNA content (H2B), while avoiding toxic/phototoxic DNA dyes. HFK-TERT cells were used in place of primary HFKs due to experimental constraints; however, sensitivity to AOH1996 was equivalent between the two cell types (Supplementary Fig. S1A). Cells were seeded in glass-bottom six-well plates at a concentration of 100,000 cells/well. Immediately before the start of the experiment, the medium was replaced with a fresh medium containing 1 μmol/L AOH1996. This dose was chosen to ensure that the compound does not elicit cell death in control cells, even at higher doses. The six-well plate was then placed on the stage of a Lionheart FX live-cell microscope (Biotek). The focus and exposure were determined automatically using software. Cells were imaged by incubation at 37°C and 5% CO_2_. Four imaging beacons were defined at suitable locations for each well. Each beacon was imaged as a 2 × 2 stitch of four individual images taken at 20× magnification at a time interval of 10 minutes for 70 hours. Postprocessing and video generation were performed using Gen5 imaging software (Biotek).

### Tubulin polymerization assay

Tubulin polymerization was measured using an optical density–based tubulin polymerization kit (Cytoskeleton, Inc., BK004P). The assay was modified from manufacturer’s instructions to ensure a concentration of 1 μmol/L for all compounds tested. The analysis includes DMSO, paclitaxel [hyperpolymerization, positive control; colcemid (inhibitor), negative control], AOH1996, and AOH-8Nq.

### Mouse xenograft study

Mice used for the initial xenograft studies (monotherapy, AOH1996 dose series, and cisplatin dose series) were obtained from a breeding colony with six breeding pairs of ES1^e^/SCID mice (RRID: MGI_8297043) and reviewed by the Institutional Animal Care and Use Committee (IACUC) at Kansas State University. The breeding pairs were donated by Dr. Philip Potter’s group at St. Jude Children’s Research Hospital (Department of Chemical Biology & Therapeutics, St. Jude Children’s Research Hospital 262 Danny Thomas Place, Memphis, TN 38105-3678). The breeding colony was subsequently expanded to 10 breeding pairs to generate mice for the HeLa and CaSki xenograft studies. As cervical cancer exclusively affects women, only female mice were used for all studies. Female pups were weaned at approximately 28 days of age. Owing to the immunocompromised nature of ES1^e^/SCID mice, all mice remained in barrier cages. As the mice were weaned, they were randomly assigned progressively to the individual study arms and then randomly assigned treatment subgroups (mock, cisplatin, AOH1996, and cisplatin in combination with AOH1996). Per IACUC protocol, a maximum of 10 mice were allotted for each treatment group. The studies were terminated early once sufficient statistical power was reached.

On day 0, ES1^e^/SCID mice that were 9 to 10 weeks of age and ranging from 16 to 24 g of weight were anesthetized with isoflurane to restrain ES1^e^/SCID mice and allow for subcutaneous xenograft implantation. The injection site was the dorsal/lateral aspect of the lower rib cage. The injections were performed using a 25-gauge 5/8′′ needle to inject 100 μL of 10^5^ HeLa or CaSki cells embedded in a 50/50 mix of PBS and Matrigel (Corning). Tumor formation was checked daily using calipers, and ES1^e^/SCID mice were observed daily for behavioral changes, as well as changes in appearance or weight. Treatment was initiated when the tumor diameters reached 5 to 6 mm. Groups 1 and 5 were administered a sterile saline injection intraperitoneally as a mock treatment once a week. Mock treatment consisted of weekly intraperitoneal saline injections combined with oral gavage of castor oil twice daily. Cisplatin was given once a week intraperitoneally, AOH1996 was given via oral gavage twice daily. Six weeks after cisplatin, AOH, or mock treatment, urine was collected, ES1^e^/SCID mice were euthanized, and tumors were removed to measure weight. ES1^e^/SCID mice were euthanized immediately if tumors reached 10% of the animal’s body weight or 15 mm in diameter or if the tumor became necrotic. Animals lost to attrition were examined by the attending veterinarian and excluded from the analysis if the cause of death was deemed unrelated to the study.

### Urinary neutrophil gelatinase-associated lipocalin assay

Mouse NGAL ELISA Kit (Crystal Chem, 80655) was used to detect urinary neutrophil gelatinase-associated lipocalin (NGAL) at the end of the xenograft study. Mouse urine was collected by gently pressing on the mouse abdomen. Mouse urine (>50 μL) was collected in a weighing tray and transferred into a microcentrifuge tube on ice. NGAL assay was conducted per the manufacturer’s instructions.

### Immunohistochemistry

Immunochemistry (IHC) was performed on Ventana Discovery Ultra IHC automated stainer (Ventana Medical Systems, Roche Diagnostics). The caPCNA rabbit polyclonal antibody was prepared and provided by R.J. Hickey (RRID: AB_3740795). A Multi Tissue Block 97-Cervix with five squamous cell carcinoma, five normal, and five dysplasia cervical tissue cases were prepared by the City of Hope pathology core. The tissue blocks were sectioned at 5 μm and put on positively charged glass slides. The slides were deparaffinized, rehydrated, and incubated with endogenous peroxidase activity inhibitor followed by antigen retrieval solution and various antibody dilutions for optimized incubation periods. The anti-caPCNA primary antibody was serially titrated in twofold dilutions from 1:25 to 1:400 and incubated at different times. The DISCOVERY anti-Rabbit HQ and DISCOVERY anti-HQ-HRP were incubated followed by staining using DISCOVERY ChromoMap DAB Kit (Roche Diagnostics) for visualization and counterstained with hematoxylin and cover-slipped. The IHC-stained slides were reviewed by the pathology core technical supervisor for test acceptability and supervising core pathologist for antigen sensitivity and staining specificity. Whole-slide images were acquired using a NanoZoomer S360 Digital Slide Scanner (Hamamatsu) and viewed by NDP.view image viewer software.

### Co-immunoprecipitation

Cells were treated with 200 nmol/L AOH1996 or AOH1996-8Nq for 24 hours, lifted and collected, washed with cold 1× PBS, and then lysed using 1 mL of 1× cell lysis buffer supplemented with protease and phosphatase inhibitor cocktails (10 μL each) per 10-cm dish. Tubes were incubated on ice for 5 minutes. Lysates were clarified by centrifugation at 14,000 × *g* for 10 minutes at 4°C. The supernatant was collected for downstream applications, and total protein concentration was quantified using the BCA assay. Lysates were diluted to 1 mg/mL in lysis buffer for pulldown assays, with aliquots reserved for input controls. For pulldown, 200 μL of protein lysate was incubated with 20 to 30 μL of preequilibrated protein A agarose beads (vortexed before use) and an appropriate dilution of PCNA rabbit primary antibody (Cell Signaling Technology, D3H8P, RRID: AB_2636979) or rabbit isotype control (Cell Signaling Technology, DA1E, RRID: AB_1550038) overnight at 4°C on a rocker. Beads were pelleted by centrifugation at 14,000 × *g* for 30 to 60 seconds, and the supernatant was discarded. Beads were washed five times with 500 μL of 1× lysis buffer, centrifuging at 14,000 × *g* for 30 to 60 seconds between washes. After the final wash, the bead pellet was resuspended in LDS sample buffer containing DTT, heated at 95°C to 100°C for 5 minutes, and centrifuged again to pellet the beads. The resulting supernatant was collected and used for SDS-PAGE.

### Ethics statement

This study was reviewed and approved by the IACUC at Kansas State University. The IACUC protocol number is 4840. All animal procedures were conducted in accordance with the guidelines of the IACUC and relevant regulatory bodies.

## Results

### PCNA and caPCNA are overexpressed in cervical cancer

**Figure 1.**
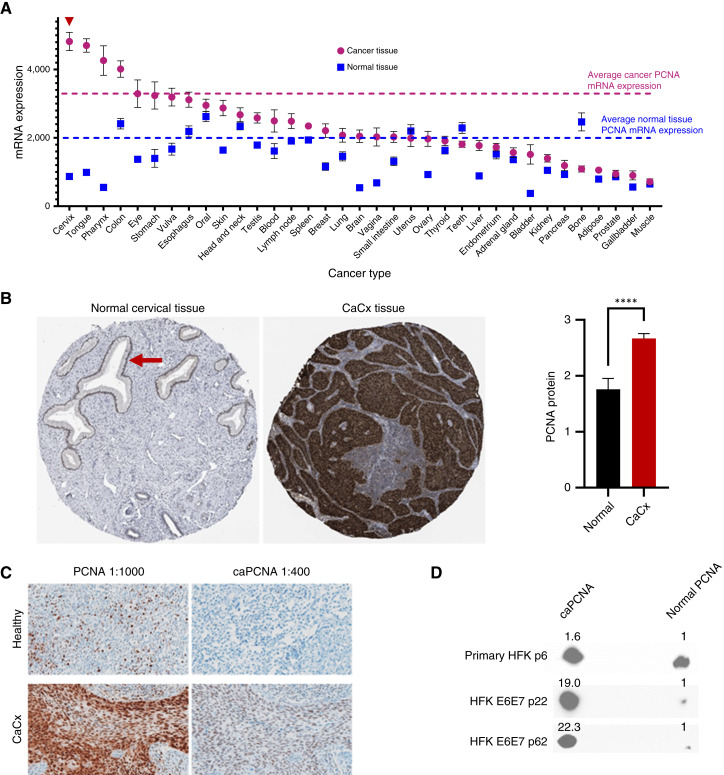
PCNA and its cancer-associated isoform caPCNA are overexpressed in cervical cancer (CaCx). **A,** PCNA mRNA expression across 35 cancer types and matched normal tissues was analyzed using the GENT2 database (34). CaCx exhibited the highest overall PCNA expression and the largest differential between tumor and normal tissues. Average PCNA mRNA expression in cancer (pink dashed line) and normal tissues (blue dashed line) are indicated. **B,** Representative IHC images from tissue microarrays showing PCNA protein expression in normal cervical tissue (left) and cervical cancer tissue (right). Scoring of PCNA staining intensity indicates significantly elevated PCNA protein levels in CaCx compared with normal tissue (bar graph inset, ****P < 0.0001). Red arrow in normal tissue indicates expected PCNA-positive basal layer. **C,** IHC staining for the normal (PC10) and cancer-associated isoform of PCNA (caPCNA) in normal and CaCx tissue with adjacent nonmalignant cervical epithelium. caPCNA is strongly expressed in tumor regions but undetectable in adjacent normal basal epithelial cells or healthy cervical epithelium. **D,** 2D gel electrophoresis of PCNA from lysates of primary HFKs and HFKs transduced with HPV16 E6E7. Signal on the left is caPCNA, and signal on the right is normal PCNA. These findings support a model in which PCNA and caPCNA are selectively upregulated in CaCx and represent candidate therapeutic targets.

We have shown that HPV E7 expression causes an increase in PCNA levels in primary epithelial cells (12). To compare the extent to which PCNA expression was elevated during cervical transformation compared with the transformation of other tissues, we performed a comprehensive analysis of PCNA expression using the GENT2 database. Of 35 comparisons, cervical cancer had the highest average PCNA mRNA expression and the most pronounced increase compared with untransformed tissue (Fig. 1A; ref. 34). IHC analysis of normal and transformed cervical tissues demonstrated a similar elevation at the protein level (Fig. 1B). Data stems from The Cancer Genome Atlas (TCGA) UID: 10409 cervical squamous cell carcinoma dataset and can be accessed via proteinatlas.org (35). To more specifically characterize caPCNA abundance, we performed IHC staining of cervical cancer tissue and adjacent nonmalignant cervical epithelium using a caPCNA-specific antibody. caPCNA was predominantly detected in malignant regions of the tissue (Fig. 1C). To define the extent to which increased caPCNA expression abundance could be attributed to HPV oncogene expression, we compared caPCNA levels in HFKs that did or did not express HPV oncogenes. (HFKs are a primary epithelial cell line commonly used to examine HPV oncogene biology because of their similarity to the cell type naturally transformed by HPV.) HPV oncogenes induce more than a 10-fold increase in the relative caPCNA abundance (Fig. 1D).

### AOH1996 selectively kills cancer cells

**Figure 2.**
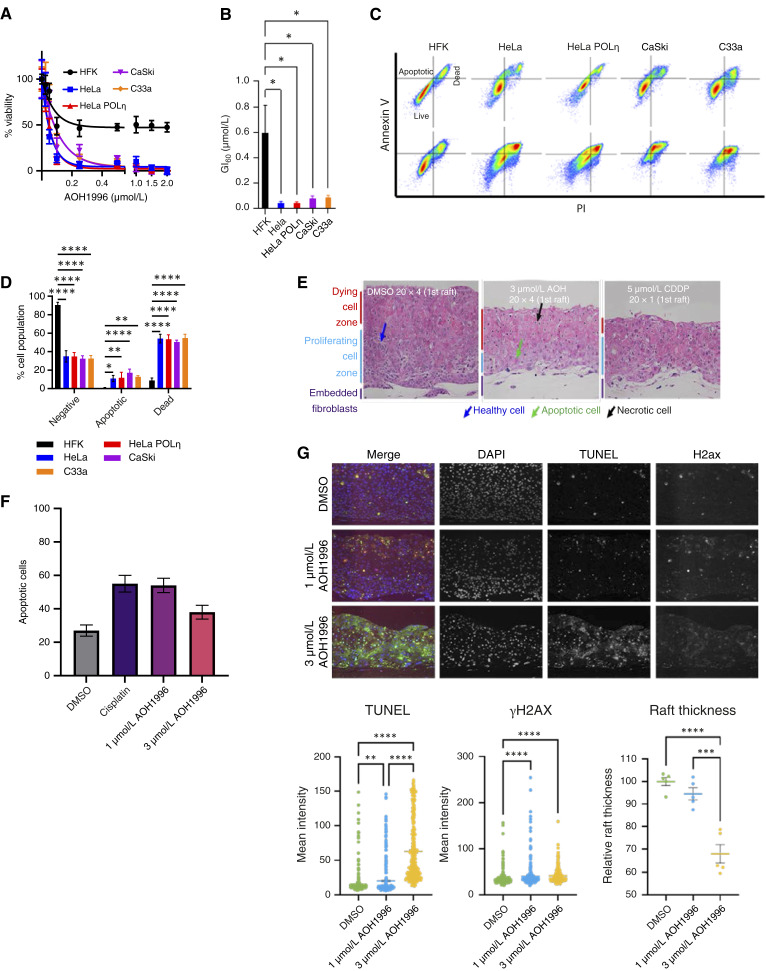
AOH1996 selectively induces apoptosis in cervical cancer (CaCx) cells. **A,** Dose–response curves of AOH1996 in primary HFKs, HeLa (HPV18^+^), CaSki (HPV16^+^), C33A (HPV^−^), and HeLa cells overexpressing POLη following 72 hours of treatment, assessed via MTT assay. HFKs show resistance, whereas all CaCx cell lines are sensitive to nanomolar concentrations. **B,** Calculated GI_50_ values confirm significantly greater sensitivity of CaCx lines to AOH1996 compared with HFKs. **C,** Representative Annexin V/PI flow cytometry plots after 48 hours of AOH1996 treatment (1 μmol/L) show live (Annexin V^−^/PI^−^), apoptotic (Annexin V^+^/PI^−^), and dead (Annexin V^+^/PI^+^ or Annexin V^−^/PI^+^) populations. **D,** Quantification of flow cytometry data indicates robust induction of apoptosis and cell death in all CaCx lines, with negligible effects in HFKs. **E,** CaSki organoid cultures treated with 3 μmol/L AOH1996 show increased cell death and reduced proliferation. Hematoxylin and eosin (H&E) staining highlights stratified epithelial structure and a dying cell zone upon AOH1996 or cisplatin treatment. **F,** Quantification of H&E-stained rafts by a pathologist. **G,** TUNEL (green) and γH2AX (red) staining indicate increased apoptosis and DNA damage in response to AOH1996. Graphs show intensity quantification and quantification of relative thickness. Data represent the mean ± SEM from at least three replicates. Statistical analysis by one-way ANOVA. *, *P* < 0.05; **, *P* < 0.01; ***, *P* < 0.001; ****, *P* < 0.0001.

Because our data suggested that AOH1996 would selectively kill cervical cancer cell lines, we conducted dose–response MTT assays on a representative panel of control and cervical cancer cell lines. HFKs served as a nonmalignant control cell line. CaSki and HeLa cells are HPV+ cervical cancer and were derived from HPV16- and HPV18-driven tumors and represent the ∼70% of cervical cancers caused by either HPV16 or HPV18. C33A cells represent the ∼10% of cervical cancer that are HPV−. Finally, HeLa cells that exogenously express polymerase eta (POLη), a translesion synthesis–associated polymerase, represent cisplatin-resistant cervical cancer (Fig. 2A). Cervical cancer cell were 7- to 14-fold more sensitive to AOH1996 than HFK cells (Fig. 2B). Increased POLη expression did not confer resistance to AOH1996 (Fig. 2A and B). The data also indicate that the HPV oncogenes can act as a sensitizer, but are not required for AOH1996 sensitivity, as C33a cells are HPV oncogene–negative. Given the differential sensitivity between primary and cervical cancer cells, we hypothesized that AOH1996 selectively induces cell death in cervical cancer cells but not in normal epithelial cells. To test this, we detected Annexin V/PI staining by flow cytometry (Fig. 2C). Consistent with our hypothesis, AOH1996 minimally increased cell death (Annexin V–positive and PI-positive) in HFK cells but caused significantly increased cell death in cervical cancer lines (Fig. 2D). Notably, Annexin V–only cell populations, indicative of early apoptosis, were small and only present in the cancer cell lines. These data suggest that AOH1996 induces cell death through a different mechanism. We next compared the efficacy of AOH1996 to cisplatin in an established organoid model of cervical cancer. The organoids were treated with 1 or 3 μmol/L AOH1996 or with 5 μmol/L cisplatin. The AOH1996 concentrations were chosen to account for transport barriers through the collagen/fibroblast plug, and 5 μmol/L of cisplatin was chosen because it is at the lower boundary for concentrations to elicit an effect. Hematoxylin and eosin staining revealed that treatment with AOH1996 and cisplatin was similarly toxic to cervical cancer organoid cultures (Fig. 2E). Specifically, 1 and 3 μmol/L of AOH1996 induced cell death of CaSki cells in cervical cancer organoids at levels similar to 5 μmol/L cisplatin (Fig. 2F). However, the pathology scoring indicates a slightly better performance of 1 μmol/L compared with 3 μmol/L, going against our expectations for dose dependency. We attributed this to the loss of raft thickness, overall cell counts, and limited number of apoptotic cells scored. We performed a TUNEL assay and immunofluorescent γH2AX staining to address this. AOH1996 increased markers of cell death and DNA damage, measured via TUNEL and γH2AX, respectively (Fig. 2G). The effects were dose-dependent and most pronounced at 3 μmol/L, in which AOH1996 increased in TUNEL straining significantly, indicating widespread cell death. These results are corroborated by a dose-dependent reduction in organoid thickness, indicating reduced proliferation and cell death [Fig. 2G (bottom right) graph].

### AOH1996 induces mitotic arrest in cervical cancer cells

**Figure 3.**
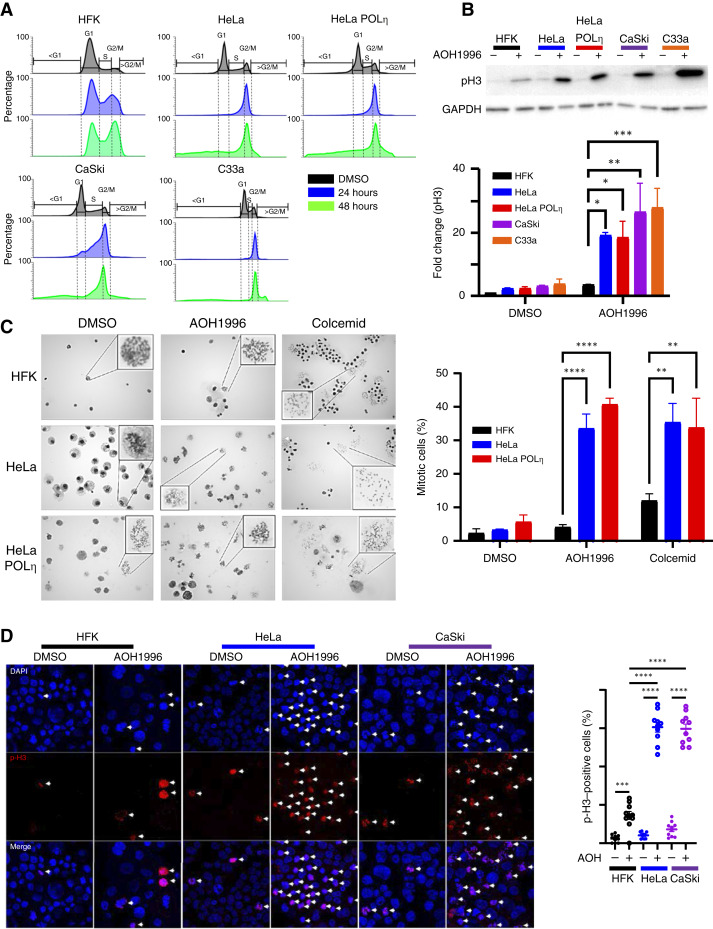
AOH1996 induces mitotic arrest in cervical cancer (CaCx) cells. **A,** Cell-cycle analysis of HFKs and CaCx cell lines (HeLa, HeLa POLη, CaSki, and C33a) treated with 1 μmol/L AOH1996 for 24 or 48 hours. DNA content was measured by PI staining and flow cytometry. AOH1996 induces a G2/M arrest at 24 hours in all cell types. At 48 hours, CaCx cells accumulate in sub-G1 and >G2 populations, indicative of apoptosis and rereplication. **B,** Immunoblot of p-H3, a marker of mitotic chromatin condensation, from lysates of cells treated with 1 μmol/L AOH1996 for 24 hours. AOH1996 significantly increases p-H3 levels in CaCx cell lines but only modestly in HFKs, indicating selective induction of mitotic arrest in transformed cells. GAPDH is used as a loading control. Bar graph shows fold change relative to DMSO-treated controls. **C,** Mitotic spread assay of HFK, HeLa, and HeLa POLη cells treated with DMSO, 1 μmol/L AOH1996, or colcemid for 24 hours. Representative brightfield images show chromatin condensation characteristic of mitosis. Insets highlight mitotic figures. Quantification (right) shows the percentage of cells in mitosis. AOH1996 induces mitotic arrest in CaCx cells but not HFKs, whereas colcemid arrests both. **D,** Confocal immunofluorescence microscopy of HeLa and CaSki cells treated with DMSO or 1 μmol/L AOH1996 for 24 hours. Cells were stained with DAPI (blue) and anti–p-H3 (red) to identify mitotic cells (white arrows). Quantification (right) shows that >60% of AOH1996-treated cells are p-H3–positive, confirming widespread mitotic arrest. Bar graphs represent the mean ± SEM from at least three independent experiments. Statistical analysis by one-way ANOVA. **, *P* < 0.01; ***, *P* < 0.001; **, *P* < 0.0001.

Because PCNA is essential for DNA replication, we assessed cell-cycle progression in our panel of cervical cancer and control cell lines after 24 and 48 hours of AOH1996 exposure. At both time points, there was an increase in HFKs in G2/M, a decrease in G1, but no other significant cell-cycle changes (Fig. 3A; Supplementary Fig. S1B–S1E). Similarly, there was an increase of CaCx cell lines in G2/M after 24 hours. However, by 48 hours, the G2/M population of cervical cancer cells gave way to elevated sub-G1 (cell death) and >G2 (unscheduled DNA synthesis) populations, an effect not seen in primary HFKs. Whereas these responses were consistent across the HPV-positive (HPV+) cervical cancer cell lines, HPV− C33a showed a slower buildup of sub-G1 cells. To determine whether AOH1996 induced a G2 or M arrest, we detected p-H3 via Western blot, as a marker of mitotic chromatin. After 24-hours of AOH1996 exposure, there was a modest increase in p-H3 in HFKs and a >10-fold increase in cervical cancer lines (Fig. 3B). These results did not notably differ by HPV status/type or cisplatin resistance/susceptibility, indicating that AOH1996 causes mitotic arrest in cervical cancer cells. We next characterized AOH1996-induced mitotic arrest using DIC microscopy to detect chromosome condensation (Fig. 3C). AOH1996 was less effective at arresting HFK cells in mitosis than an established spindle poison, colcemid. In contrast, AOH1996 and colcemid induced similar levels of mitotic arrest in HeLa and HeLa POLη cells. These findings demonstrate that AOH1996-induced mitotic arrest is selective for transformed epithelial cells and does not broadly disrupt mitotic entry in untransformed cells. We found further evidence that AOH1996 selectively induces mitotic arrest in HeLa and CaSki cells by detecting a mitotic marker (p-H3) via immunofluorescent microscopy (Fig. 3D).

### AOH1996 causes mitotic death in cervical cancer cells

**Figure 4.**
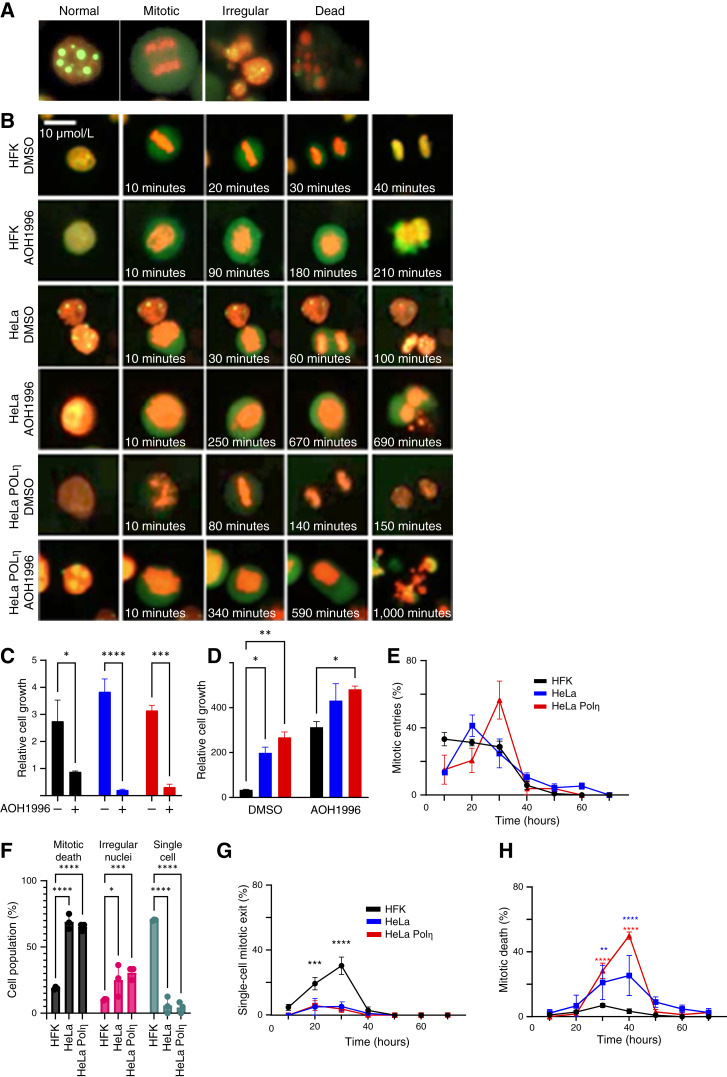
AOH1996 induces mitotic arrest and mitotic death in cervical cancer cells. **A,** Representative images of nuclear phenotypes observed by live-cell imaging of HFK, HeLa, and HeLa POLη cells stably expressing GFP-LaminB1 (green) and mCherry-H2B (red). Cells were classified as normal, mitotic, irregular, or dead based on nuclear envelope integrity and chromatin morphology. Scale bars = 5 μm. **B,** Time-lapse microscopy of cells treated with 1 μmol/L AOH1996 or DMSO control. HFK, HeLa, and HeLa POLη cells undergo timely mitotic progression in control conditions. AOH1996 treatment causes extended mitotic arrest and death, particularly in transformed cells. Scale bars = 10 μm. **C,** Relative cell growth over a 72-hour imaging window. AOH1996 halts proliferation in HFKs and leads to significant cell loss in HeLa and HeLa POLη cells. **D,** Quantification of time spent in mitosis. AOH1996 significantly prolongs mitosis in all lines, with the greatest delay observed in HeLa POLη cells. **E,** Time-course of mitotic entry following AOH1996 treatment. All cell types enter mitosis at similar rates. **F,** Analysis of mitotic outcomes. HFKs primarily undergo single-cell mitotic exit (∼71%), whereas mitotic death is the predominant fate in HeLa and HeLa POLη cells (∼70% to 74%). **G,** Temporal distribution of single-cell mitotic exits. Most events occur between 20 and 30 hours after treatment in HFKs. **H,** Temporal profile of mitotic death. HeLa and HeLa POLη cells undergo mitotic death between 30 and 50 hours after treatment. Data represent the mean ± SEM from three independent experiments. Statistical analysis was performed using one-way or two-way ANOVA. *, *P* < 0.05; **, *P* < 0.01; ***, *P* < 0.001; ****, *P* < 0.0001.

To better define the relationship between AOH1996-induced mitotic arrest and cell death, we performed live-cell imaging of HFK, HeLa, and HeLa POLη cells expressing GFP-tagged LaminB1 (nuclear membrane marker) and mCherry-tagged histone H2B (DNA marker). This system enabled us to classify mitotic outcomes (normal, prolonged, irregular, and death) in AOH1996-treated cells (Fig. 4A and B; Supplementary Fig. S2A). All mock-treated cell lines proliferated steadily over the 72-hour imaging period (Fig. 4C). In HFK cells, AOH1996 halted proliferation, but the number of HKFs also did not decrease. In contrast, AOH1996 caused a marked reduction in cervical cancer cell viability. AOH1996 did not significantly alter mitotic entry but caused a prolonged mitosis in each cell line (Fig. 4D and E). This delay was prolonged in cervical cancer cells (Fig. 4E). We next defined the fate of AOH1996-treated cells exiting mitosis (Fig. 4F). Most untransformed HFKs exited mitosis as a single cell with decondensed chromatin and a reformed nuclear envelope. In contrast, most cervical cancer cells died after exiting mitosis without a reformed nuclear membrane or decondensed chromatin. There was also an increased proportion of cervical cancer cells that exited mitosis with irregular nuclei. Fixed-cell microscopy provided higher resolution images of cells with irregular nuclei, showing they had characteristics of mitotic stress, like multilobular nuclei (Supplementary Fig. S2B and S2C). Further live-cell imaging analysis demonstrated that cervical cancer cells with irregular nuclei either died shortly after forming irregular nuclei or reentered mitosis and subsequently died during a second attempt to undergo mitosis (Fig. 4F). We then characterized the temporal relationship between mitotic exit as a single cell and mitotic death. Most HFK cells exited mitosis as single cells between 20 to 30 hours after treatment (Fig. 4G). Cervical cancer cells died after bypassing this checkpoint, undergoing mitotic death 10 to 20 hours later (Fig. 4H). These results suggest the anticancer activity of AOH1996 is in part due to its ability to exclusively cause cervical cancer cells to attempt to complete mitosis in a setting in which this leads to mitotic death.

### AOH1996 disrupts mitosis by interfering with centrosome and spindle organization

**Figure 5.**
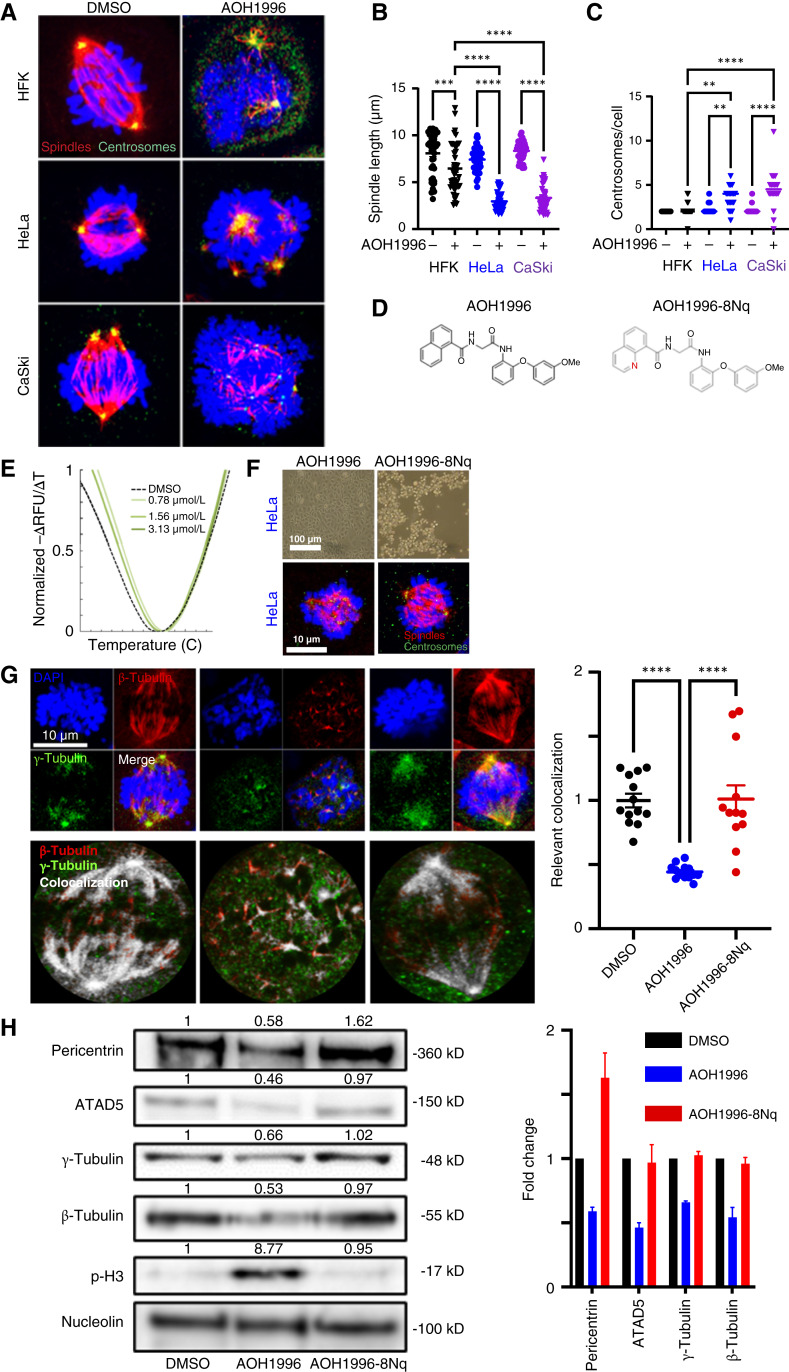
AOH1996 disrupts centrosome organization and spindle integrity. **A,** Representative immunofluorescence microscopy images of HFK, HeLa, and CaSki cells treated with DMSO or 1 μmol/L AOH1996 for 24 hours, stained for β-tubulin (red), γ-tubulin (green), and DNA (DAPI, blue). In DMSO-treated cells, mitotic spindles and centrosomes appear organized and bipolar. In contrast, AOH1996-treated cells exhibit multipolar, shortened, and disorganized spindles with supernumerary centrosomes. **B,** Quantification of spindle length shows a significant reduction in AOH1996-treated cells across all lines, confirming spindle collapse or failure. **C,** Quantification of centrosome number per cell reveals a significant increase following AOH1996 treatment, most pronounced in cervical cancer (CaCx) cells. **D,** Chemical structures of AOH1996 and its structurally related analog AOH1996-8Nq, which contains a nitrogen substitution in the aromatic ring and lacks cytotoxic or mitosis-disrupting activity. **E,** Protein thermal shift assay of recombinantly expressed 6xHis PCNA with AOH1996-8Nq. A PCNA-stabilizing shift of 0.5°C at each concentration indicates binding interaction. **F,** Brightfield and immunofluorescence microscopy images of HeLa cells treated with 200 nmol/L AOH1996 or AOH1996-8Nq. AOH1996 causes cell rounding and centrosome/spindle disruption, whereas AOH1996-8Nq does not. **G,** Immunofluorescence microscopy of β-tubulin (red), γ-tubulin (green), and DAPI (blue) in HeLa cells. AOH1996 significantly decreases colocalization between γ-tubulin and β-tubulin, as shown by quantification (right). AOH1996-8Nq has no effect. Bottom, pixel-based colocalization maps show overlapping γ/β-tubulin signal (white). **H,** Western blot analysis of centrosome components (pericentrin and γ-tubulin), spindle protein (β-tubulin), and mitotic marker (p-H3) from HeLa cells treated with DMSO, AOH1996, or AOH1996-8Nq. AOH1996 decreases centrosomal protein levels and increases p-H3 abundance. AOH1996-8Nq does not alter these levels. Nucleolin serves as a loading control. Bar graph represents corresponding quantification. Bar graphs represent the mean ± SEM from three independent experiments. Statistical comparisons were performed using two-way ANOVA. ****, *P* < 0.0001; ***, *P* < 0.001; **, *P* < 0.0001.

Because PCNA interacts with the tubulin cytoskeleton (36), we hypothesized that AOH1996 abrogates mitosis by hindering centrosome integrity and spindle architecture. To test this, we visualized mitotic spindles (β-tubulin) and centrosomes (γ-tubulin) following AOH1996 treatment. All mock-treated mitotic HFKs had two distinct centrosomes and elongated bipolar spindles (Fig. 5A). Consistent with published data (37), some mitotic cervical cancer cells had three or more centrosomes but generally had spindles radiating from two poles. AOH1996 caused spindles to become shortened, disorganized, or absent in all cell lines. The spindles also frequently lacked a recognizable centrosomal polarity. These features are consistent with centrosome dysfunction and impaired microtubule nucleation. To quantify spindle defects, we measured the length of individual mitotic spindles. AOH1996 significantly reduced spindle length in all cell types (Fig. 5B). AOH1996 also increased the number of centrosomes in HFKs and cervical cancer cells. However, AOH1996 induced more centrosomes in cervical cancer than HFKs (Fig. 5C). To investigate structure–activity relationships, we designed and synthesized AOH1996-8Nq, in which 1-naphthyl group was replaced by its bioisosteres, 8-quionolyl group, for use as a negative control (Fig. 5D). AOH1996-8Nq retains the same size and shape as AOH1996, and thermal shift assay shows it retains its ability to interact with PCNA (Fig. 5E). AOH1996-8Nq did not induce mitotic arrest [Fig. 5F (top): rounded mitotic cells when treated with AOH1996 and flat, adherent cells when treated with AOH1996-8Nq] or centrosomal abnormalities [Fig. 5F (bottom) row]. AOH1996 disrupts centrosome–spindle coupling, as measured by γ-tubulin and β-tubulin colocalization (Fig. 5G), whereas AOH1996-8Nq failed to reduce γ/β-tubulin colocalization. Furthermore, AOH1996 decreased the protein abundance of the centrosomal and spindle components γ-tubulin, β-tubulin, ATAD5, and pericentrin, indicating a broad disruption of the centrosomal protein complex (Fig. 5H; refs. 36, 38). These data identify structural components of AOH1996 that are important for its selective cytotoxicity and shows that interference with spindle assembly and centrosome function are significant contributors to AOH1996-mediated cytotoxicity in cervical cancer cells.

### AOH1996 disrupts tubulin dynamics and reduces PCNA–γ-tubulin interaction in cancer cells

**Figure 6.**
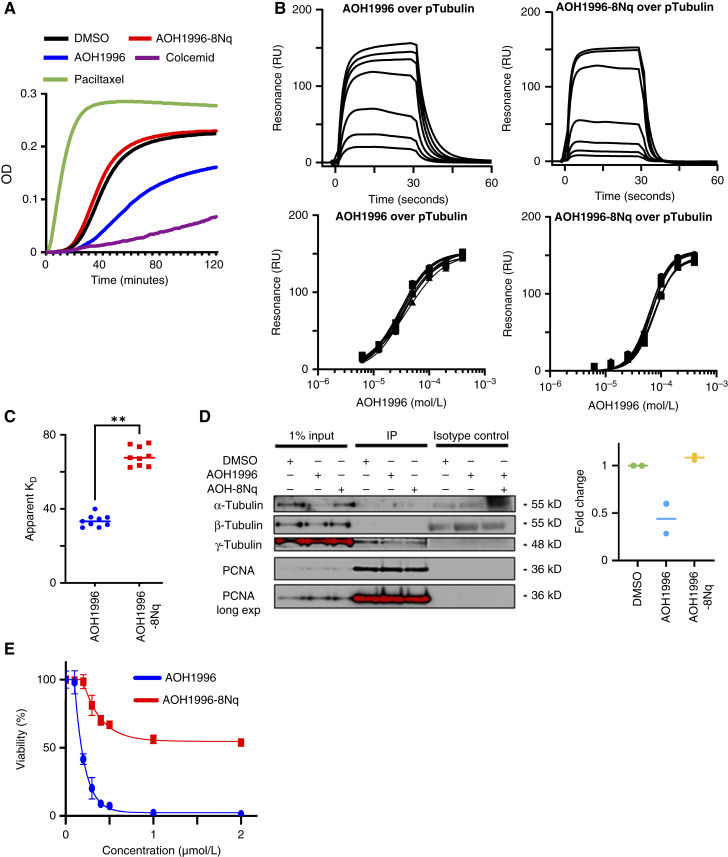
AOH1996 binds to tubulin and disrupts PCNA–γ-tubulin interactions. **A,** Tubulin polymerization assay performed *in vitro* using purified tubulin. AOH1996 partially inhibits polymerization relative to DMSO and paclitaxel (positive control) but more effectively than the inert analog AOH1996-8Nq and the microtubule-depolymerizing agent colcemid (negative control). **B,** SPR analysis of AOH1996 and AOH1996-8Nq binding to purified tubulin. Sensorgrams (top) and fitted binding curves (bottom) demonstrate that AOH1996 binds tubulin with higher affinity than AOH1996-8Nq. **C,** Apparent dissociation constants (Kd) derived from SPR data. AOH1996 binds tubulin with a mean Kd of ∼30 μmol/L, whereas AOH1996-8Nq shows modest, yet significantly weaker, binding with a mean Kd of ∼70 μmol/L. Graphs represent the mean and range of nine independent experiments. **D,** Co-immunoprecipitation (co-IP) of endogenous PCNA from HeLa lysates treated with or without AOH1996 or AOH1996-8Nq, followed by immunoblotting for α-, β-, or γ-tubulin. AOH1996 treatment reduces the interaction between PCNA and γ-tubulin by ∼50%, whereas AOH1996-8Nq has no effect. Normal rabbit IgG was used as a negative IP control. Right, quantification of γ-tubulin co-IP signal normalized to DMSO. Bar graphs represent the mean and range from at least two biological replicates. **E,** Dose–response curve from MTT assay shows reduced impact of AOH1996-8Nq (IC_50_ value not reached) on HeLa viability compared with AOH1996-treated (IC_50_ = 125 nmol/L) cells. Statistical comparisons were made using unpaired two-tailed *t* tests. **, *P* < 0.01.

We used a cell-free tubulin polymerization assay to determine whether AOH1996 directly interferes with microtubule polymerization. AOH1996 inhibited tubulin polymerization without the presence of PCNA, indicating a PCNA-independent ability to disrupt tubulin polymerization (Fig. 6A). This inhibition was significantly less robust than traditional spindle poisons. To define the extent to which AOH1996 directly binds tubulin, we conducted SPR with purified tubulin protein. Consistent with its ability to disrupt tubulin polymerization, AOH1996 modestly bound tubulin (Fig. 6B and C). AOH1996-8Nq:tubulin binding was weaker than AOH1996:tubulin binding. AOH1996-8Nq also had no detectable impact on tubulin polymerization. These findings suggest that AOH1996 can directly engage tubulin and partially disrupt polymerization. However, the modest binding affinity and intermediate polymerization inhibition do not fully account for the potent antimitotic activity observed in cancer cells. We next tested the impact of AOH1996 on interactions between PCNA and γ-tubulin. Immunoprecipitation of PCNA from mock and treated HeLa lysates revealed that AOH1996, but not AOH1996-8Nq, reduces the interaction between PCNA and γ-tubulin (Fig. 6D). We also found no evidence of interaction between PCNA and α- or β-tubulin. Together, these data suggest that disruption of the PCNA–γ-tubulin interaction is critical to AOH1996’s function as a mitotic disruptor. Consistent with this, AOH1996-8Nq, which retains PCNA-binding activity, exhibits reduced but not completely lost cytotoxicity compared with AOH1996. However, AOH1996-8Nq does not reach the IC_50_ value threshold within the tested concentration range (Fig. 6E). Collectively, these findings demonstrate that interference with the caPCNA–γ-tubulin interaction is a key contributor to the anticancer activity of AOH1996.

### AOH1996 synergizes with cisplatin *in vitro*

**Figure 7.**
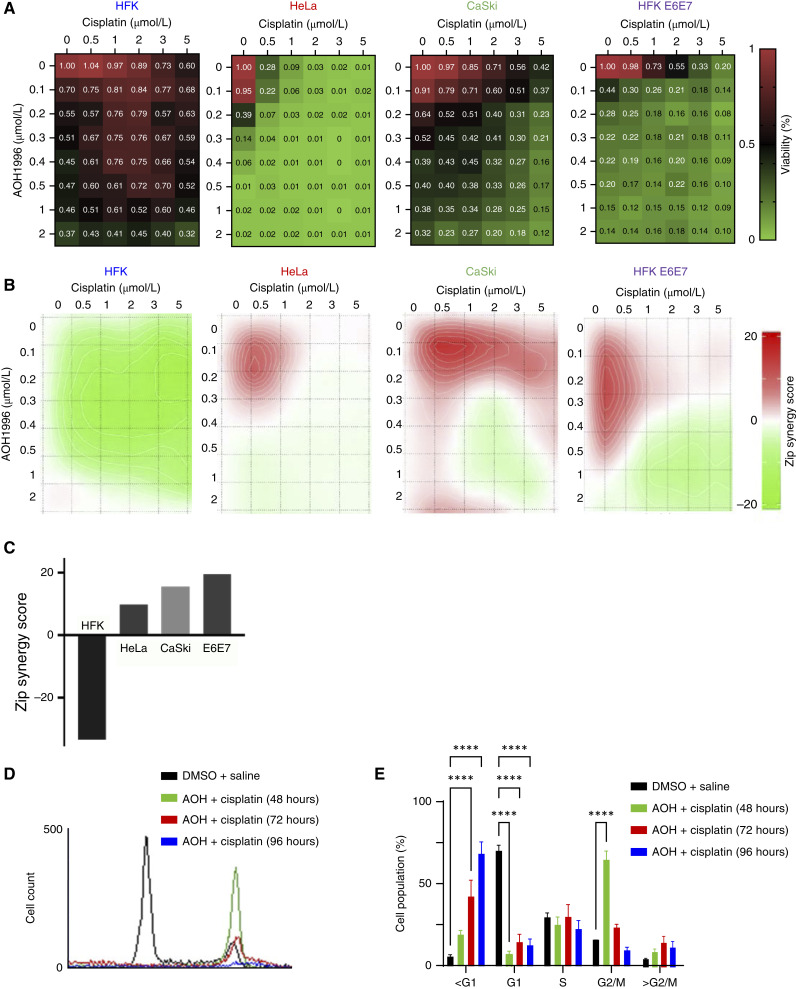
AOH1996 synergizes with cisplatin to enhance cytotoxicity in cervical cancer cells. **A,** Dose–response matrix of AOH1996 and cisplatin combinations in HFKs, HeLa, and CaSki cells measured by MTT viability assay. Heatmaps show normalized cell viability after 96 hours of treatment. Green indicates reduced viability, and red indicates higher survival. **B,** Synergy contour maps generated from matrix data in **A**, analyzed using SynergyFinder 3.0. A synergy score >10 indicates synergy, and <−10 indicates antagonism. **C,** AOH1996 and cisplatin were strongly synergistic in HeLa, CaSki, and HFK 16E6E7 cells antagonistic in HFKs. Table denotes absolute synergy scores. **D,** Flow cytometry histogram of HeLa cells treated with 1 μmol/L AOH1996 followed by 1 μmol/L cisplatin at indicated time points. Increased sub-G1 peak at 72 and 96 hours indicates enhanced cell death. **E,** Quantification of cell cycle profiles confirms that AOH1996 pretreatment followed by cisplatin induces a G2/M arrest at 48 hours and a progressive increase in the sub-G1 population at 72 and 96 hours, consistent with apoptotic cell death. Bar graphs show the mean ± SEM from three independent experiments. Statistical analysis was performed using two-way ANOVA. ****, *P* < 0.0001.

Because DNA damage is more toxic to cells progressing through mitosis (39) and that only cervical cancer cells progressed through mitosis after AOH1996 exposure, we hypothesized that AOH1996 specifically sensitizes cervical cancer cells to cisplatin. Supporting this hypothesis, we found synergy between AOH1996 and cisplatin in cervical cancer cell lines and primary cells expressing HPV oncogenes exposed to a gradient of AOH1996 and cisplatin concentrations (Fig. 7A–C). This demonstrated that HPV oncogene expression alone can be sufficient to drive sensitivity and synergy [Fig. 7A (bottom)] but does not exclude other modes of sensitization. We predicted that optimal synergy between the drugs would require treatment with AOH1996 followed by cisplatin so that cervical cancer cells are passing through mitosis when they experience cisplatin-induced DNA damage. We began testing this hypothesis by confirming that cisplatin arrested cervical cancer cells in S-phase (Supplementary Fig. S3A–S3C). Then, we treated cells with a gradient of AOH1996 for 24 hours followed by a gradient of cisplatin for 72 hours. Primary HFKs did not show significant sensitivity to this combination, and drug synergy analysis shows significant antagonism (Fig. 7B and C). In contrast, cervical cancer cells were sensitive to the combination treatment, and AOH1996 synergized with cisplatin (Fig. 7B and C). Notably, expression of the HPV 16 E6/E7 oncogenes in primary HFKs is sufficient to render the cells sensitive to the drug combination in a synergistic manner (Fig. 7A–C). Pretreatment with AOH1996 followed by cisplatin resulted in a robust G2/M arrest and increased cell death (Fig. 7D and E, increase in the sub-G1 population). Reversing the treatment order resulted in an S-phase arrest and reduced synergy (Supplementary Fig. S4A–S4D).

### AOH1996 allows cisplatin to effectively manage cervical cancer at lower, less toxic doses

**Figure 8.**
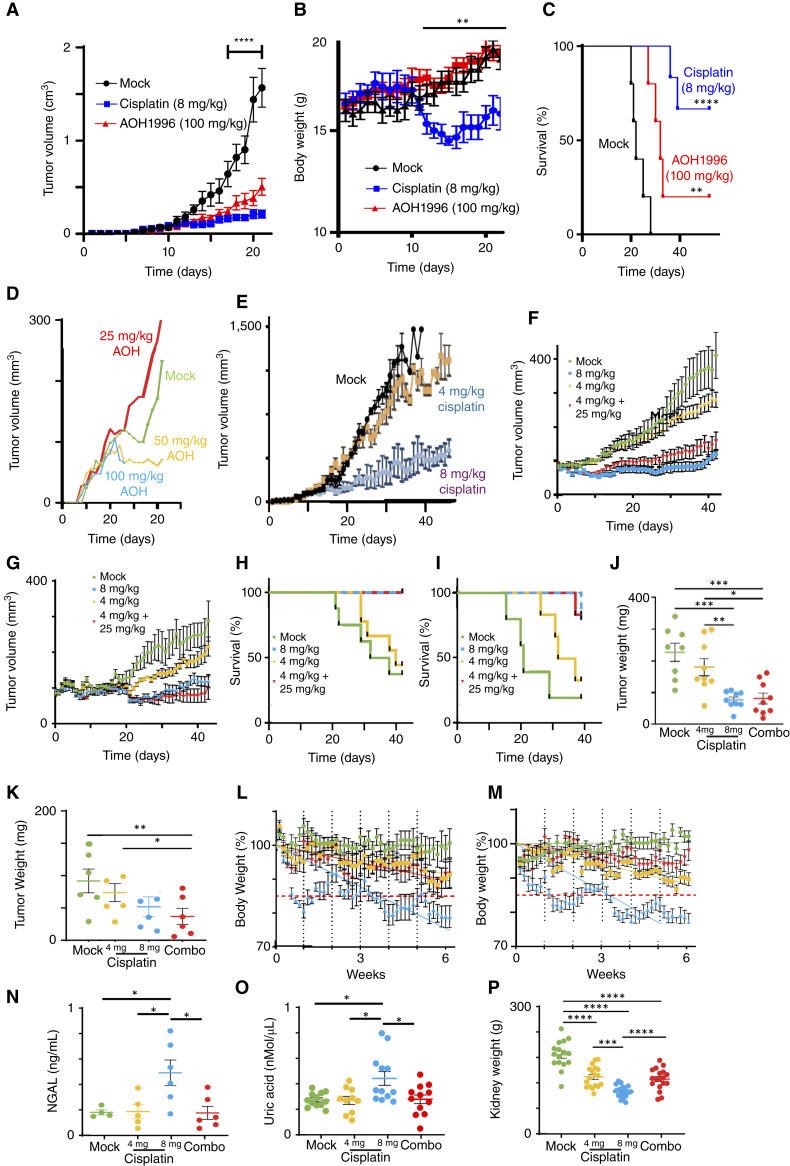
AOH1996 synergizes with cisplatin *in vivo* and reduces cisplatin-associated toxicity. **A,** HeLa xenograft tumor volumes after monotherapy with mock treatment (black), cisplatin (blue), or AOH1996 (red). **B**, Corresponding mouse body weights. **C**, Kaplan–Meier survival analysis of mice treated with vehicle control, 8 mg/kg cisplatin (blue), or 100 mg/kg AOH1996 (red). Both AOH1996 and cisplatin significantly reduce tumor growth and extend survival, though cisplatin is more effective. **D,** Volume analysis of HeLa xenografts treated with varying doses of AOH1996 reveals that doses below 50 mg/kg exhibit limited monotherapy efficacy. **E,** Determination of subtherapeutic cisplatin dose. HeLa xenograft volumes of mock (black), subtherapeutic 4 mg/kg (beige), and therapeutic 8 mg/kg (gray) are shown. **F,** HeLa xenograft volume measurements in mice treated with vehicle, therapeutic-dose cisplatin (8 mg/kg), subtherapeutic-dose cisplatin (4 mg/kg), or the combination of 25 mg/kg AOH1996 and 4 mg/kg cisplatin. **G,** CaSki xenograft volume measurements in mice treated with vehicle, therapeutic-dose cisplatin (8 mg/kg), subtherapeutic-dose cisplatin (4 mg/kg), or the combination of 25 mg/kg AOH1996 and 4 mg/kg cisplatin. **H,** Survival analysis of HeLa xenograft–bearing mice. Cutoff set at 290 mm^3^. **I,** Survival analysis of CaSki xenograft–bearing mice. **J,** Final tumor weights at necropsy from HeLa xenografts. **K,** Final tumor weights at necrophsy from CaSki xenografts. **L,** Relative body weights of HeLa xenograft–bearing mice throughout treatment. **M,** Relative body weights of CaSki xenograft–bearing mice throughout treatment. **N,** Urinary NGAL, a marker of kidney injury, levels in mice treated as indicated. **O,** Uric acid levels in mice treated as shown. **P,** Kidney weight at necrophsy in mice treated as indicated. Data represent the mean ± SEM from at least eight mice per group. Statistical comparisons were made using two-way ANOVA for tumor volumes and NGAL levels and log-rank (Mantel–Cox) tests for survival. *, *P* < 0.05; **, *P* < 0.01; ***, *P* < 0.001; ****, *P* < 0.0001.

We next determined the efficacy of AOH1996 *in vivo* as a single agent in a HeLa xenograft model. Oral administration of 100 mg/kg AOH1996 (twice daily by oral gavage) significantly reduced tumor volume and improved survival compared with vehicle-treated controls (Fig. 8A–C; Supplementary Fig. S5A and S5B). It also has a notably lower systemic toxicity, indicated by mouse body weight retention in comparison with cisplatin (Fig. 8B). However, AOH1996 was less effective than weekly intraperitoneal injections of cisplatin at 8 mg/kg. We readily observed cisplatin systemic toxicity characterized by weight loss, dehydration, and lack of grooming. This toxicity required us to skip weekly cisplatin doses at least once in each cisplatin-treated mouse. AOH1996 was well-tolerated, causing no overt signs of toxicity throughout treatment. We hypothesized that AOH1996 could reduce the toxicity associated with cisplatin by synergizing and lowering its effective dose. To test this hypothesis, we identified subtherapeutic doses for each agent, defined as the highest dose that was well-tolerated but had no significant effect on tumor growth (Fig. 8D and E). Then, we determined whether the combination of subtherapeutic cisplatin and AOH1996 could control HeLa and CaSki xenograft growth (Fig. 8F–M). The combination of subtherapeutic cisplatin and AOH1996 doses reduced tumor volume and weight and improved survival to levels comparable with animals treated with a therapeutic dose of cisplatin (Fig. 8F–K; Supplementary Fig. S5C). The combination therapy was markedly better tolerated than a therapeutic dose of cisplatin in terms of weight loss (Fig. 8L and M), skipped doses, dehydration, and lack of grooming. We hypothesized that cisplatin-induced weight loss was due to kidney damage. Cisplatin’s adverse effect on kidneys is one among several factors that can result in weight loss and dehydration (40). Nephrotoxicity was assessed at the tissue level via postmortem histochemical analysis of kidneys and urinary NGAL and uric acid levels detected during treatment. NGAL and uric acid levels were elevated in mice treated with a therapeutic dose of cisplatin. In contrast, mice treated with the combination of subtherapeutic doses of AOH1996 and cisplatin had levels indistinguishable from vehicle controls (Fig. 8N and O), suggesting preserved kidney function. We next assessed kidney atrophy by weighing excised kidneys (Fig. 8P). A therapeutic dose of cisplatin reduced kidney mass more than mock treatment or the combination of subtherapeutic doses of cisplatin and AOH1996. IHC analysis of these tumors found rare overt signs of nephrotoxicity (acute tubular epithelial cell injury, karyomegaly, and protein casts in collecting ducts) that were similarly common among treatment groups, suggesting that our efforts to manage cisplatin-induced toxicity (e.g., injections of lactated Ringer’s solution for dehydration treatment) were successful (Supplementary Fig. S6A–S6C). Together, these results demonstrate that the combination of subtherapeutic AOH1996 and cisplatin reduced xenograft tumor burden with less weight loss and lower NGAL levels than high-dose cisplatin.

## Discussion

Our detailed molecular analysis of AOH1996-induced changes to cervical cancer cells resulted in several novel findings that are important to consider during the continued clinical development of AOH1996 (phase 1 chemorefractory solid tumor trial, NCT05227326). For example, the mitotic defects observed in AOH1996-treated cervical cancer cells include supernumerary centrosomes, disorganized spindles, and nuclear abnormalities indicative of mitotic catastrophe, a form of cell death frequently seen in response to spindle assembly checkpoint failure. The vulnerability of cervical cancer cells to AOH1996-induced mitotic stress likely arises from their altered centrosome homeostasis and reliance on tightly regulated mitotic progression—features that are common across many cancer types (41). This suggests that AOH1996 derives specificity not only by binding to caPCNA but also from its ability to cause mitotic stress that transformed cells manage poorly. This sensitivity can be driven by HPV oncogenes, but other nonviral oncogene drivers undoubtably exist and should be identified in future studies.

In cervical cancer xenograft models, AOH1996 allowed a subtherapeutic dose of cisplatin to control tumor growth as effectively as a therapeutic dose of cisplatin. This allowed mice to avoid the toxicity associated with cisplatin treatment (e.g., weight loss and nephrotoxicity). We conclude that AOH1996 could be used in combination with cisplatin to improve cervical cancer care. Clinically, low doses of cisplatin are given prior to radiation as a sensitizing agent. Our data suggest that the addition of AOH1996 to this treatment regimen would likely increase the efficacy of cisplatin as an adjuvant for radiotherapy. Given that both cisplatin and radiation induce cytotoxicity by causing DNA damage, AOH1996 may also allow radiotherapy to be effective at lower doses. The potential synergy between AOH1996 and radiotherapy needs to be explored in future experiments.

We found that the order cervical cancer cells are exposed to AOH1996 and cisplatin affects the synergy between the drugs. Pretreatment with AOH1996 sensitized cervical cancer cells to subsequent cisplatin exposure, leading to enhanced cell death and a shift in cell-cycle dynamics toward mitotic death. However, these effects were less striking when cisplatin exposure occurred first. Our data suggest that pretreatment with cisplatin prevented AOH1996-mediated mitotic death. These findings indicate that the sequence of treatment should be carefully considered in future combination regimens. Our AOH1996:cisplatin cotreatment analysis also suggests that AOH1996 may protect untransformed cells from cisplatin-associated toxicity, as it behaves antagonistically. Thus, AOH1996 may reduce cisplatin side effects by lowering the therapeutic dose and by protecting untransformed cells.

AOH1996 causes cytotoxicity in cervical cancer cells by inducing aberrant mitosis. Whereas traditional antimitotic drugs can result in undesirable side effects, we observe no evidence of AOH1996-associated toxicity. The development of AOH1996-8Nq provides a valuable negative control for continual assessment of this possibility. AOH1996-8Nq, despite its close structural similarity to AOH1996, does not induce mitotic disruption or anticancer activity, highlighting the importance of specific structural features for the biological effects of AOH1996. Moreover, if future clinical testing of AOH1996 were to reveal unexpected toxicity related to mitotic defects, the existence of a well-characterized analog like AOH1996-8Nq would be instrumental for refining therapeutic strategies. Conversely, if AOH1996-induced mitotic disruption proves clinically beneficial, rational design of next-generation analogs could further enhance its ability to target mitosis selectively, enhancing cancer cell specificity.

AOH1996 monotherapy was less effective at managing tumor size and promoting survival than cisplatin. The reduced antitumor effect of orally dosed AOH1996 relative to intraperitoneal cisplatin may reflect differences in pharmacokinetics or exposure associated with the route of administration, although these parameters were not directly assessed here and require further evaluation. Yet, AOH1996 may have utility as a second-wave treatment for cervical cancer. We found that it effectively killed a panel of cervical cancer cell lines *in vitro*, chosen to represent more than 80% of cervical cancer. In xenografts, AOH1996 was effective as a monotherapy and very well tolerated at a high dose. It was also effective at killing cisplatin-resistant cervical cancer cells. These data suggest that AOH1996 could represent an alternative treatment option for cervical cancer that has acquired or innate resistance to cisplatin. Notably, a combination of subtherapeutic doses of AOH1996 (25 mg/kg) and cisplatin (4 mg/kg) achieved the same tumor control efficacy as a therapeutic dose of cisplatin (8 mg/kg) while exhibiting less therapy-associated side effects. These xenograft findings suggest that AOH1996 may enable effective tumor control at reduced cisplatin doses, potentially limiting systemic and nephrotoxicity, although dedicated pharmacokinetic and safety studies will be needed to define clinically relevant dosing strategies. Furthermore, whereas these xenograft studies demonstrate robust tumor control with reduced cisplatin-associated toxicity, they were conducted in immunodeficient hosts, limiting the assessment of immune contributions to therapeutic response. Future studies could leverage immunocompetent syngeneic models such as Tal3, an HPV16 E6/E7–driven cervicovaginal carcinoma model (42), or the widely used U14 cervical carcinoma model (43), to evaluate treatment effects in the context of host immunity. Cisplatin has been shown to influence antitumor immunity through DNA damage–associated innate immune signaling and altered immune-cell recruitment, although these effects can be context-dependent (44). Emerging evidence suggests that AOH1996 treatment may also be compatible with, or potentially enhance, antitumor immune infiltration (45). Evaluation of the AOH1996–cisplatin combination in syngeneic models would therefore clarify whether immune mechanisms contribute to the observed therapeutic window and could inform rational integration with immunotherapeutic treatment regimens. Lastly, AOH1996 effectively killed primary cells expressing HPV oncogenes, a system that more closely resembles pre-cancerous lesions than cervical cancer. This suggests that AOH1996 could be developed to treat premalignant cervical lesions, with further potential as a broader antiviral. These findings support further evaluation of AOH1996 in preclinical and clinical studies.

## Supplementary Material

Supplemental Figure 1 AOH1996 induces G2/M arrest followed by apoptosis in cervical cancer cells.

Supplemental Figure 2 AOH1996 induces abnormal nuclear morphology resulting in cell death in cervical cancer cells.

Supplemental Figure 3 Cisplatin induces S-phase arrest and delayed apoptosis in HeLa cells.

Supplemental Figure 4 Cisplatin pretreatment impairs AOH1996 efficacy by blocking mitotic entry in cervical cancer cells.

Supplemental Figure 5 Xenograft Sizes, Study Arms and Treatment Regimen.

Supplemental Figure 6 H&E Analysis Did Not Detect Gross Deferential Nephrotoxicity.

## Data Availability

Data from GENT2 are freely available online at http://gent2.appex.kr (34). The PCNA IHC data are available from TCGA UID: 10409, https://datacatalog.mskcc.org/dataset/10409. Additionally, all data, images, and videos are available upon request to the corresponding author.
