# Supplementary material for: Targeting Cancer-Associated PCNA with AOH1996 Induces Mitotic Catastrophe and Enhances Cisplatin Therapy in Cervical Cancer
Source: Cancer Res Commun. 2026 May 27;6(5):1220–38. doi: 10.1158/2767-9764.CRC-25-0648 (PMC13213708; doi:10.1158/2767-9764.CRC-25-0648)
Supplement: Supplemental Figure 1 — AOH1996 induces G2/M arrest followed by apoptosis in cervical cancer cells. [file crc-25-0648_supplemental_figure_1_suppsf1.pptx]

## Slide 1
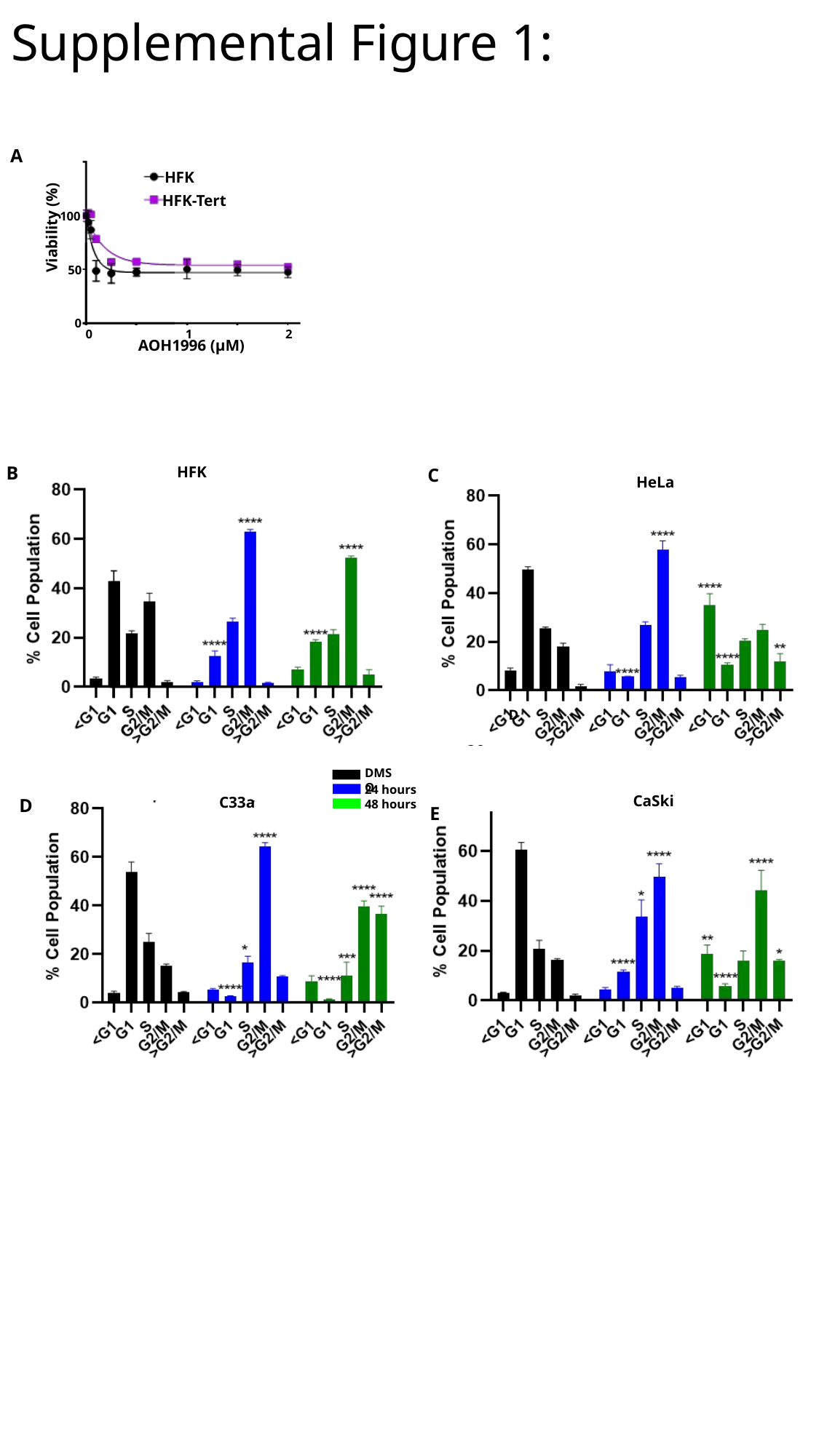

# Supplemental Figure 1:
A
HFK
100
Viability (%)
50
0
HFK-Tert
0
1
2
AOH1996 (μM)
B
HFK
C
HeLa
D
DMSO
24 hours
48 hours
CaSki
C33a
D
E

## Slide 2
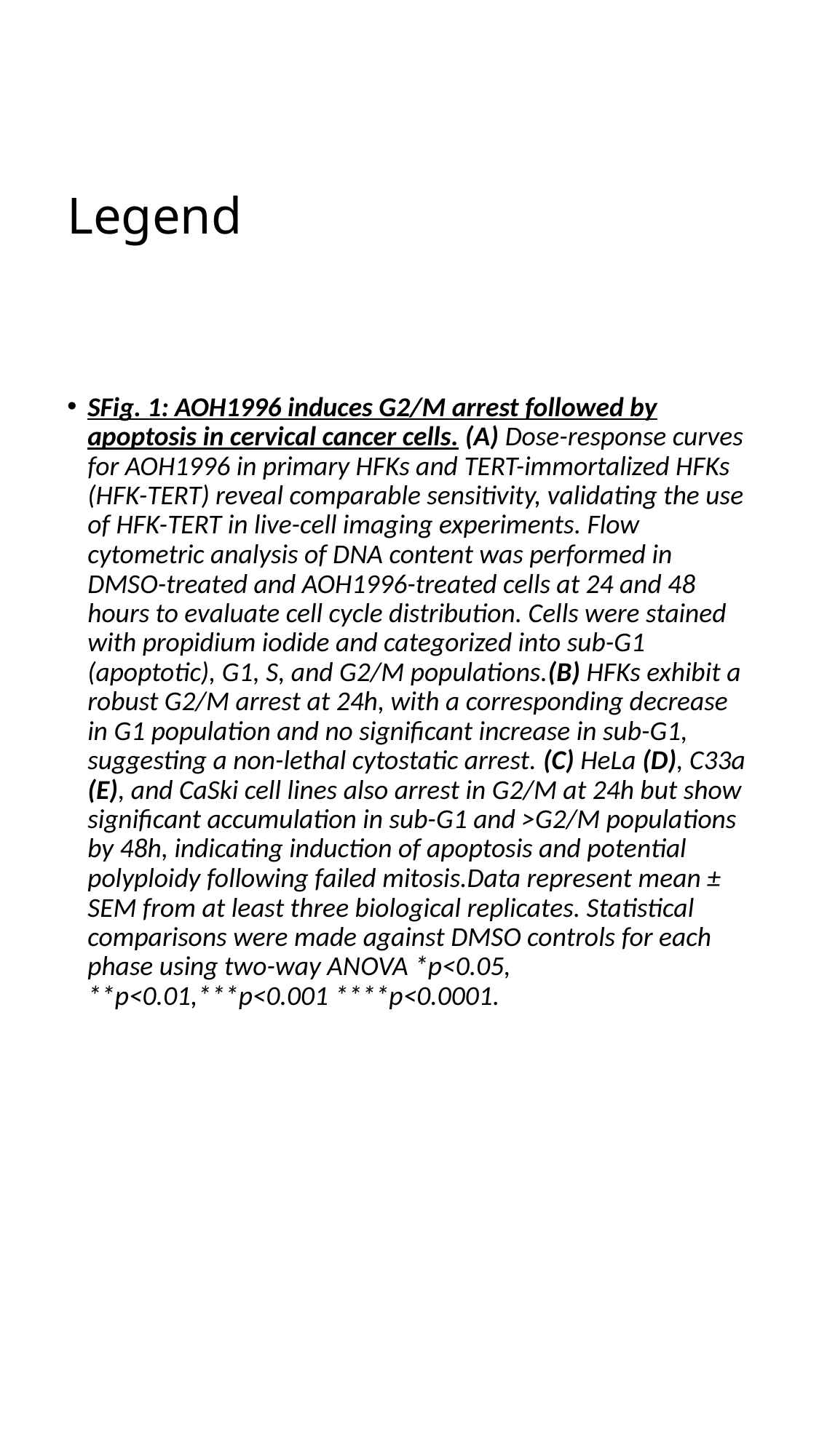

# Legend
SFig. 1: AOH1996 induces G2/M arrest followed by apoptosis in cervical cancer cells. (A) Dose-response curves for AOH1996 in primary HFKs and TERT-immortalized HFKs (HFK-TERT) reveal comparable sensitivity, validating the use of HFK-TERT in live-cell imaging experiments. Flow cytometric analysis of DNA content was performed in DMSO-treated and AOH1996-treated cells at 24 and 48 hours to evaluate cell cycle distribution. Cells were stained with propidium iodide and categorized into sub-G1 (apoptotic), G1, S, and G2/M populations.(B) HFKs exhibit a robust G2/M arrest at 24h, with a corresponding decrease in G1 population and no significant increase in sub-G1, suggesting a non-lethal cytostatic arrest. (C) HeLa (D), C33a (E), and CaSki cell lines also arrest in G2/M at 24h but show significant accumulation in sub-G1 and >G2/M populations by 48h, indicating induction of apoptosis and potential polyploidy following failed mitosis.Data represent mean ± SEM from at least three biological replicates. Statistical comparisons were made against DMSO controls for each phase using two-way ANOVA *p<0.05, **p<0.01,***p<0.001 ****p<0.0001.
