# Supplementary material for: Targeting Cancer-Associated PCNA with AOH1996 Induces Mitotic Catastrophe and Enhances Cisplatin Therapy in Cervical Cancer
Source: Cancer Res Commun. 2026 May 27;6(5):1220–38. doi: 10.1158/2767-9764.CRC-25-0648 (PMC13213708; doi:10.1158/2767-9764.CRC-25-0648)
Supplement: Supplemental Figure 2 — AOH1996 induces abnormal nuclear morphology resulting in cell death in cervical cancer cells. [file crc-25-0648_supplemental_figure_2_suppsf2.pptx]

## Slide 1
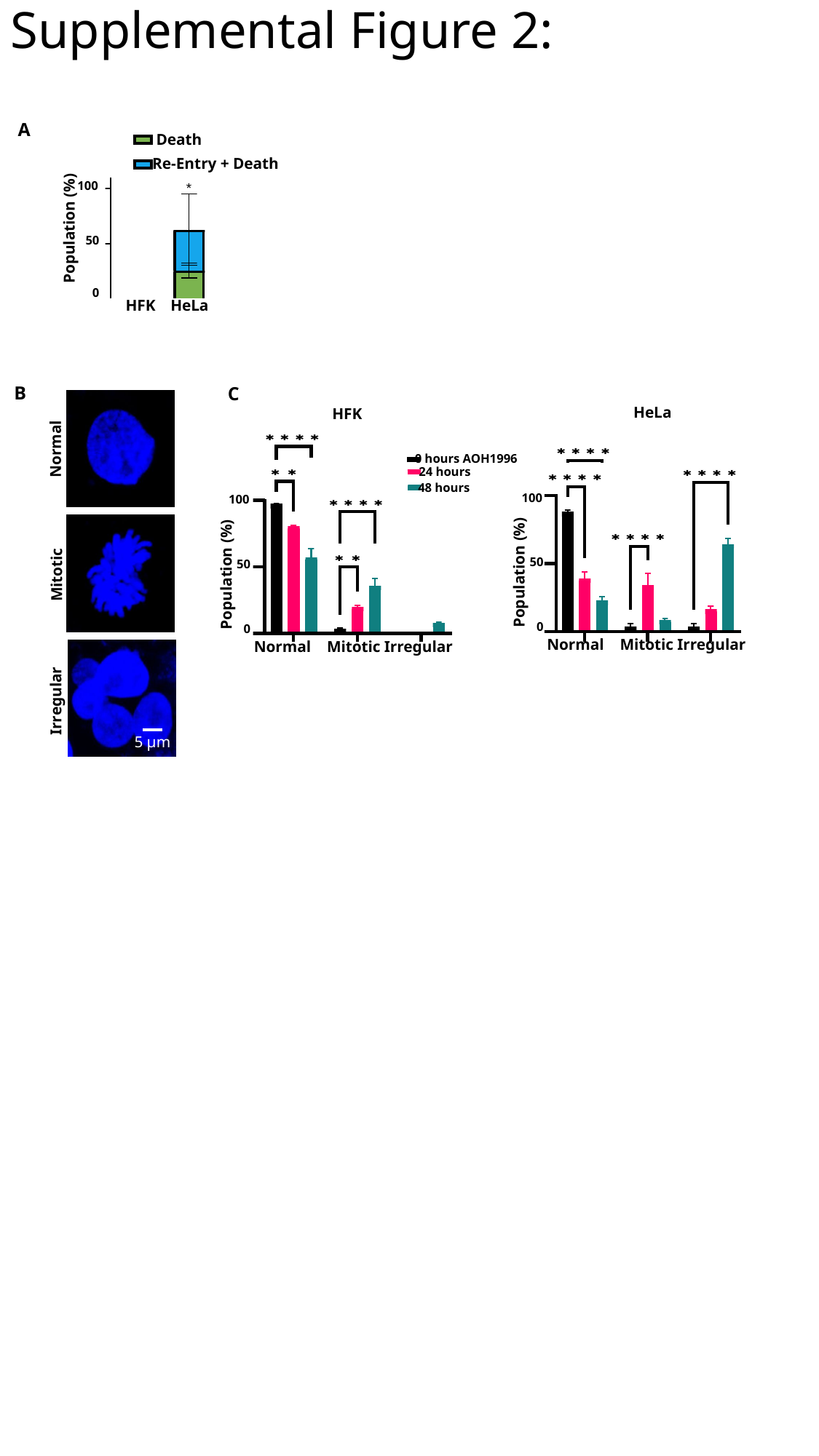

Supplemental Figure 2:
A
Death
Re-Entry + Death
100
Population (%)
50
0
HFK
HeLa
B
C
HeLa
HFK
100
50
Population (%)
0
Irregular
Normal
Mitotic
100
50
Population (%)
0
Irregular
Normal
Mitotic
Normal
0 hours AOH1996
24 hours
48 hours
Mitotic
Irregular
5 μm

## Slide 2
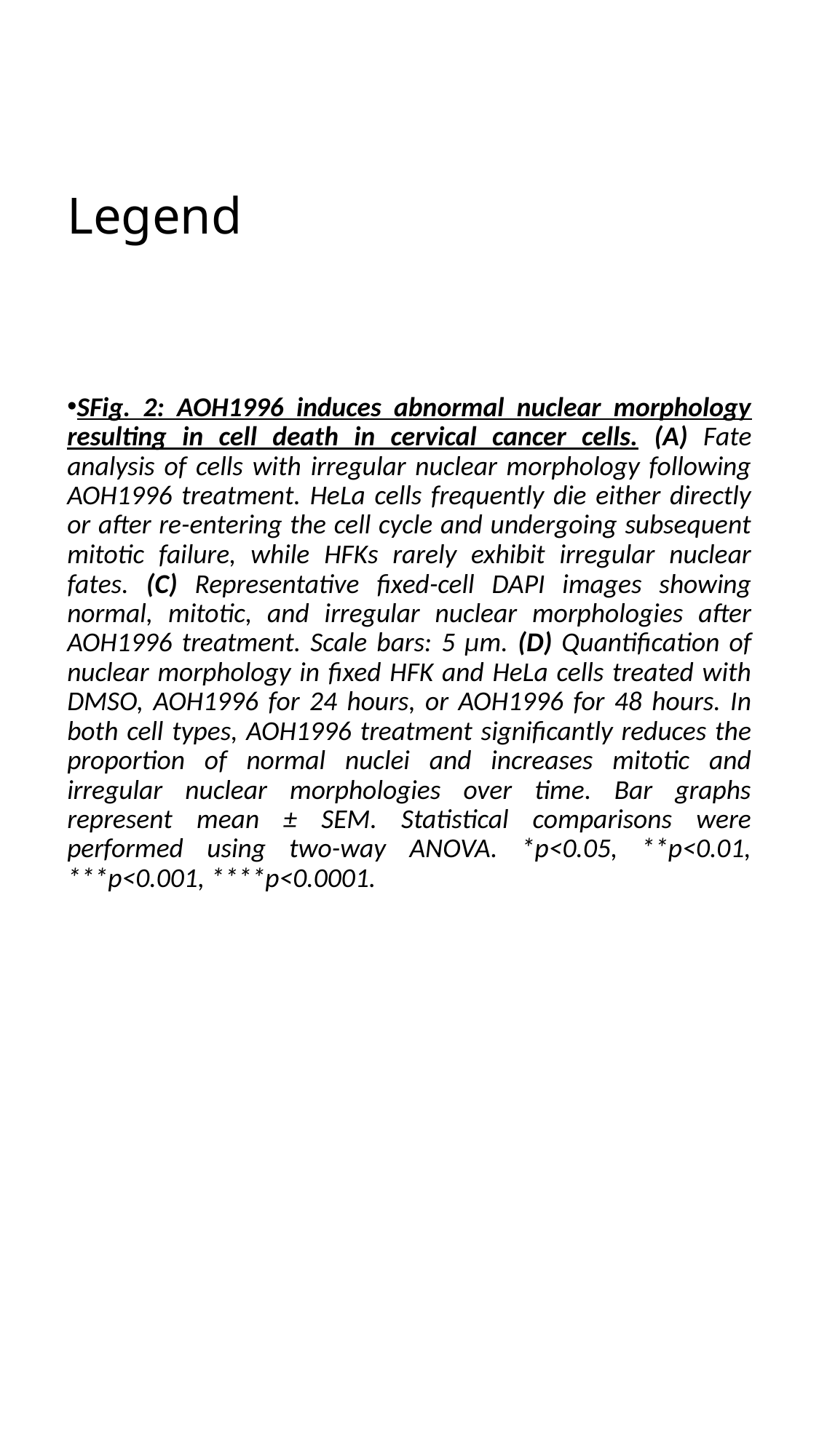

# Legend
SFig. 2: AOH1996 induces abnormal nuclear morphology resulting in cell death in cervical cancer cells. (A) Fate analysis of cells with irregular nuclear morphology following AOH1996 treatment. HeLa cells frequently die either directly or after re-entering the cell cycle and undergoing subsequent mitotic failure, while HFKs rarely exhibit irregular nuclear fates. (C) Representative fixed-cell DAPI images showing normal, mitotic, and irregular nuclear morphologies after AOH1996 treatment. Scale bars: 5 μm. (D) Quantification of nuclear morphology in fixed HFK and HeLa cells treated with DMSO, AOH1996 for 24 hours, or AOH1996 for 48 hours. In both cell types, AOH1996 treatment significantly reduces the proportion of normal nuclei and increases mitotic and irregular nuclear morphologies over time. Bar graphs represent mean ± SEM. Statistical comparisons were performed using two-way ANOVA. *p<0.05, **p<0.01, ***p<0.001, ****p<0.0001.
