# Supplementary material for: Targeting Cancer-Associated PCNA with AOH1996 Induces Mitotic Catastrophe and Enhances Cisplatin Therapy in Cervical Cancer
Source: Cancer Res Commun. 2026 May 27;6(5):1220–38. doi: 10.1158/2767-9764.CRC-25-0648 (PMC13213708; doi:10.1158/2767-9764.CRC-25-0648)
Supplement: Supplemental Figure 3 — Cisplatin induces S-phase arrest and delayed apoptosis in HeLa cells. [file crc-25-0648_supplemental_figure_3_suppsf3.pptx]

## Slide 1
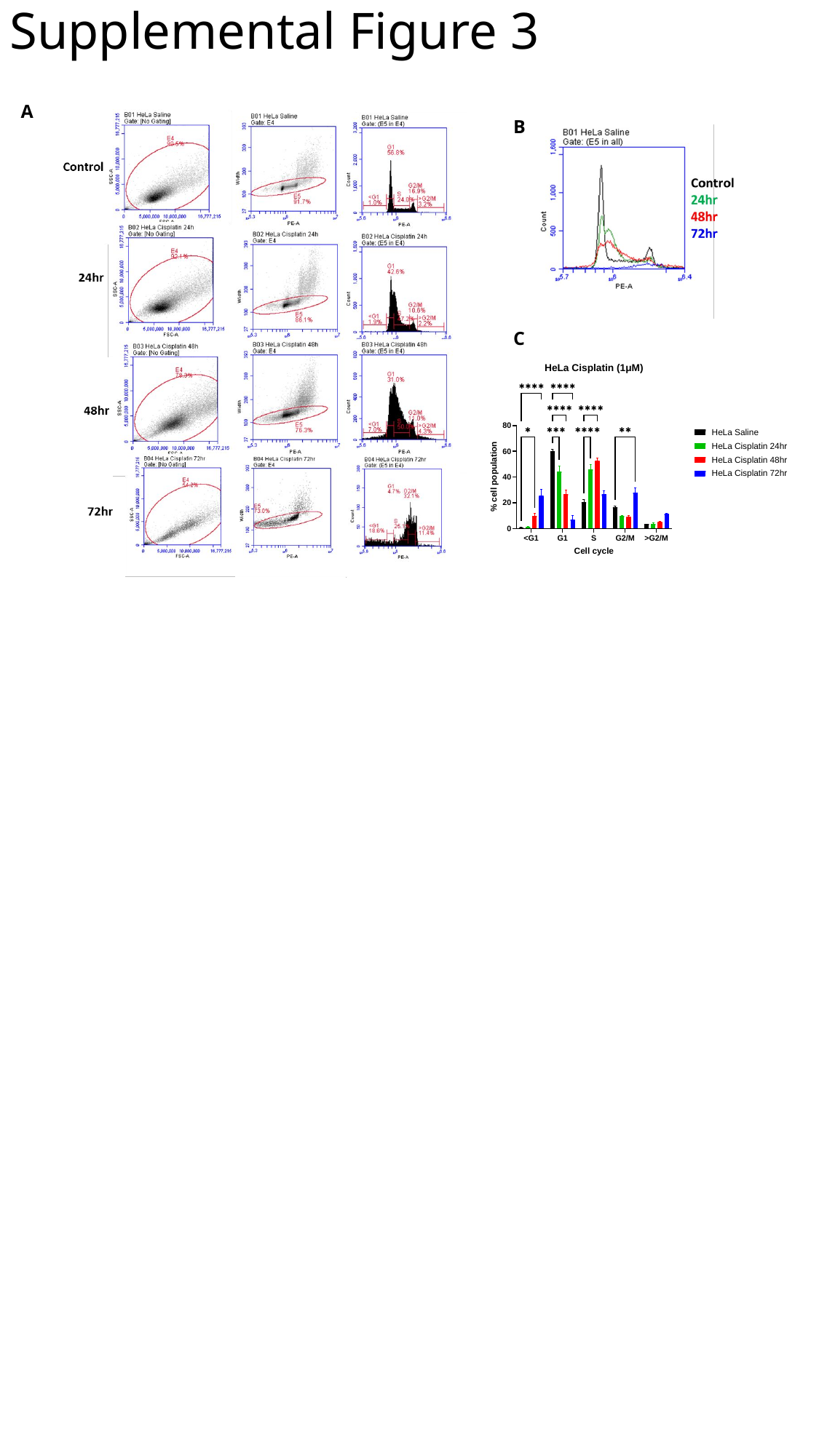

# Supplemental Figure 3
A
B
C

## Slide 2
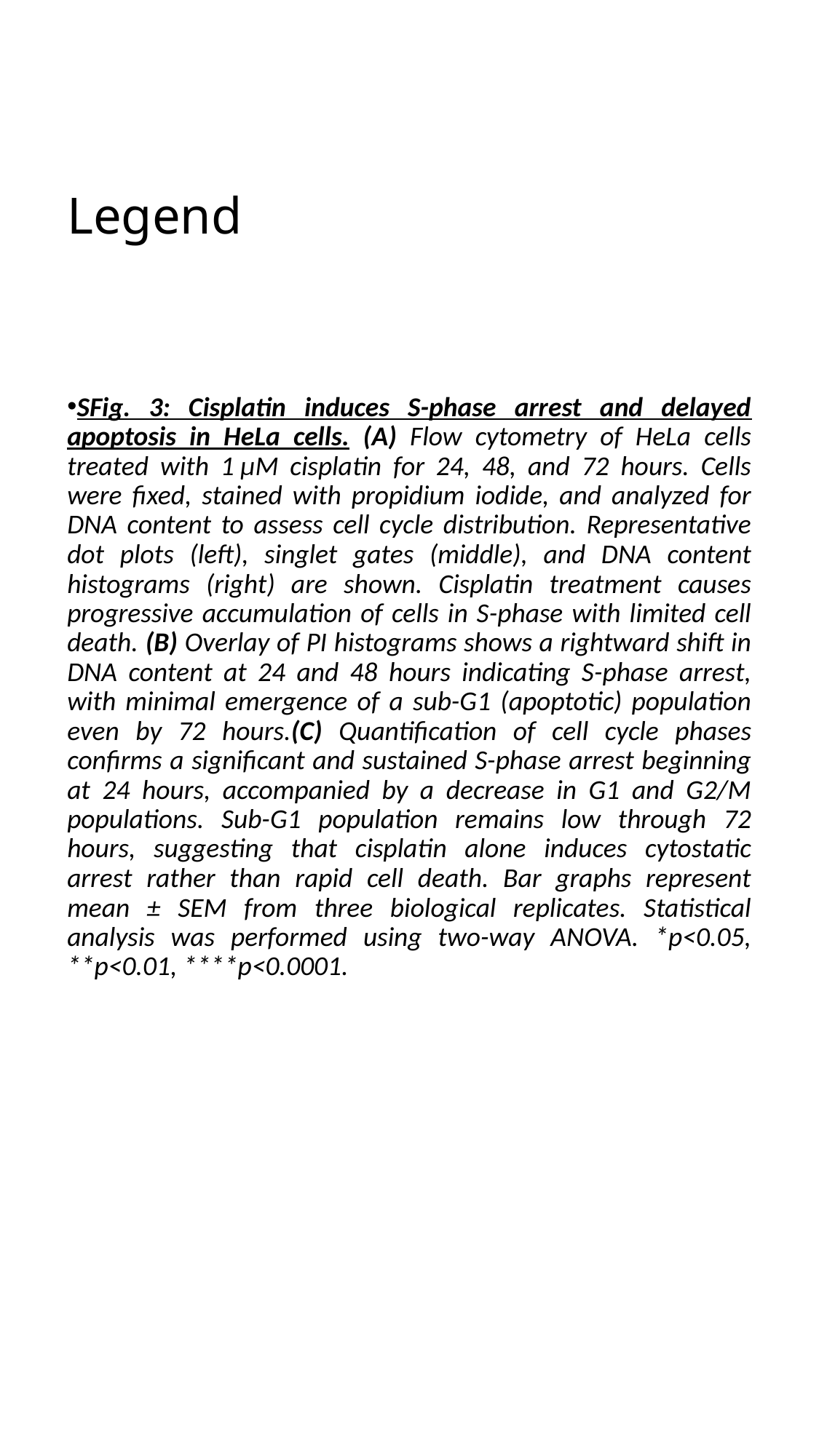

# Legend
SFig. 3: Cisplatin induces S-phase arrest and delayed apoptosis in HeLa cells. (A) Flow cytometry of HeLa cells treated with 1 μM cisplatin for 24, 48, and 72 hours. Cells were fixed, stained with propidium iodide, and analyzed for DNA content to assess cell cycle distribution. Representative dot plots (left), singlet gates (middle), and DNA content histograms (right) are shown. Cisplatin treatment causes progressive accumulation of cells in S-phase with limited cell death. (B) Overlay of PI histograms shows a rightward shift in DNA content at 24 and 48 hours indicating S-phase arrest, with minimal emergence of a sub-G1 (apoptotic) population even by 72 hours.(C) Quantification of cell cycle phases confirms a significant and sustained S-phase arrest beginning at 24 hours, accompanied by a decrease in G1 and G2/M populations. Sub-G1 population remains low through 72 hours, suggesting that cisplatin alone induces cytostatic arrest rather than rapid cell death. Bar graphs represent mean ± SEM from three biological replicates. Statistical analysis was performed using two-way ANOVA. *p<0.05, **p<0.01, ****p<0.0001.
