# Supplementary material for: Targeting Cancer-Associated PCNA with AOH1996 Induces Mitotic Catastrophe and Enhances Cisplatin Therapy in Cervical Cancer
Source: Cancer Res Commun. 2026 May 27;6(5):1220–38. doi: 10.1158/2767-9764.CRC-25-0648 (PMC13213708; doi:10.1158/2767-9764.CRC-25-0648)
Supplement: Supplemental Figure 4 — Cisplatin pretreatment impairs AOH1996 efficacy by blocking mitotic entry in cervical cancer cells. [file crc-25-0648_supplemental_figure_4_suppsf4.pptx]

## Slide 1
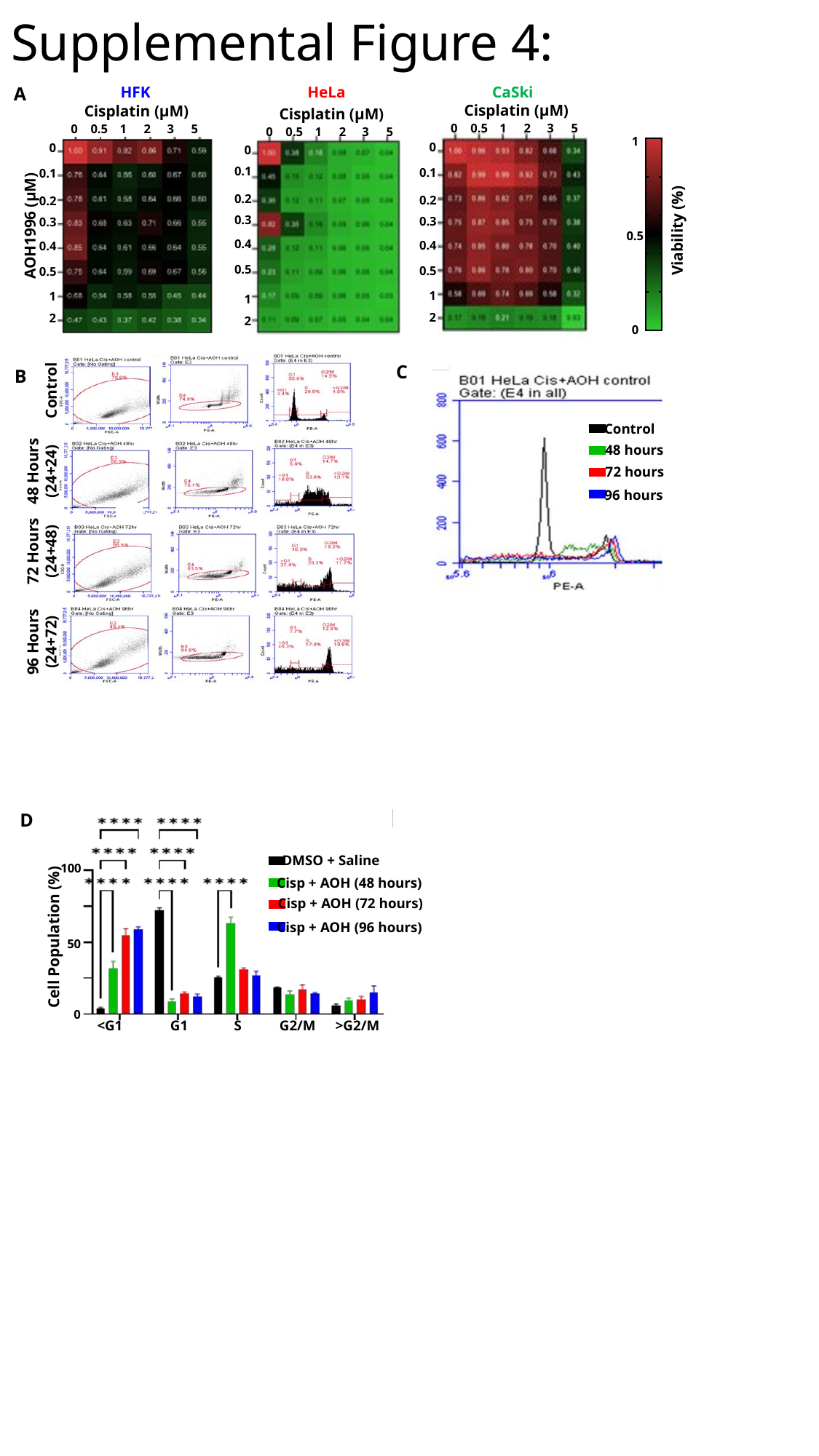

# Supplemental Figure 4:
A
HFK
HeLa
CaSki
Cisplatin (μM)
Cisplatin (μM)
Cisplatin (μM)
0
0.5
1
2
3
5
0
0.5
1
2
3
5
0
0.5
1
2
3
5
1
0
0
0
0.1
0.1
0.1
0.2
0.2
0.2
0.3
0.3
0.3
AOH1996 (μM)
Viability (%)
0.5
0.4
0.4
0.4
0.5
0.5
0.5
1
1
1
2
2
2
0
C
B
Control
Control
48 hours
72 hours
96 hours
48 Hours
(24+24)
72 Hours
(24+48)
96 Hours
(24+72)
D
DMSO + Saline
Cisp + AOH (48 hours)
Cisp + AOH (72 hours)
Cisp + AOH (96 hours)
100
Cell Population (%)
50
0
<G1
G1
S
G2/M
>G2/M

## Slide 2
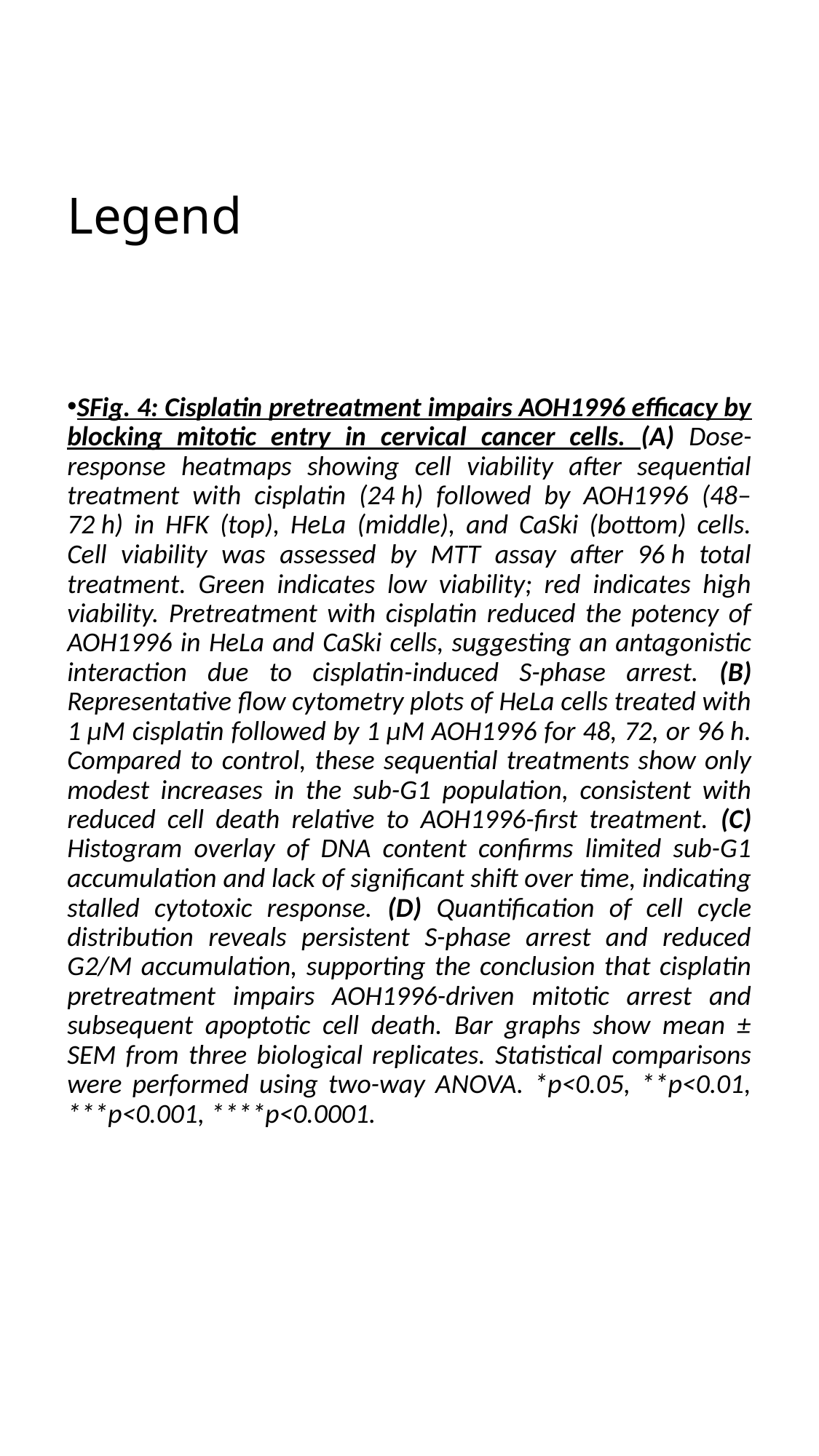

# Legend
SFig. 4: Cisplatin pretreatment impairs AOH1996 efficacy by blocking mitotic entry in cervical cancer cells. (A) Dose-response heatmaps showing cell viability after sequential treatment with cisplatin (24 h) followed by AOH1996 (48–72 h) in HFK (top), HeLa (middle), and CaSki (bottom) cells. Cell viability was assessed by MTT assay after 96 h total treatment. Green indicates low viability; red indicates high viability. Pretreatment with cisplatin reduced the potency of AOH1996 in HeLa and CaSki cells, suggesting an antagonistic interaction due to cisplatin-induced S-phase arrest. (B) Representative flow cytometry plots of HeLa cells treated with 1 μM cisplatin followed by 1 μM AOH1996 for 48, 72, or 96 h. Compared to control, these sequential treatments show only modest increases in the sub-G1 population, consistent with reduced cell death relative to AOH1996-first treatment. (C) Histogram overlay of DNA content confirms limited sub-G1 accumulation and lack of significant shift over time, indicating stalled cytotoxic response. (D) Quantification of cell cycle distribution reveals persistent S-phase arrest and reduced G2/M accumulation, supporting the conclusion that cisplatin pretreatment impairs AOH1996-driven mitotic arrest and subsequent apoptotic cell death. Bar graphs show mean ± SEM from three biological replicates. Statistical comparisons were performed using two-way ANOVA. *p<0.05, **p<0.01, ***p<0.001, ****p<0.0001.
