# Supplementary material for: Targeting Cancer-Associated PCNA with AOH1996 Induces Mitotic Catastrophe and Enhances Cisplatin Therapy in Cervical Cancer
Source: Cancer Res Commun. 2026 May 27;6(5):1220–38. doi: 10.1158/2767-9764.CRC-25-0648 (PMC13213708; doi:10.1158/2767-9764.CRC-25-0648)
Supplement: Supplemental Figure 5 — Xenograft Sizes, Study Arms and Treatment Regimen. [file crc-25-0648_supplemental_figure_5_suppsf5.pptx]

## Slide 1
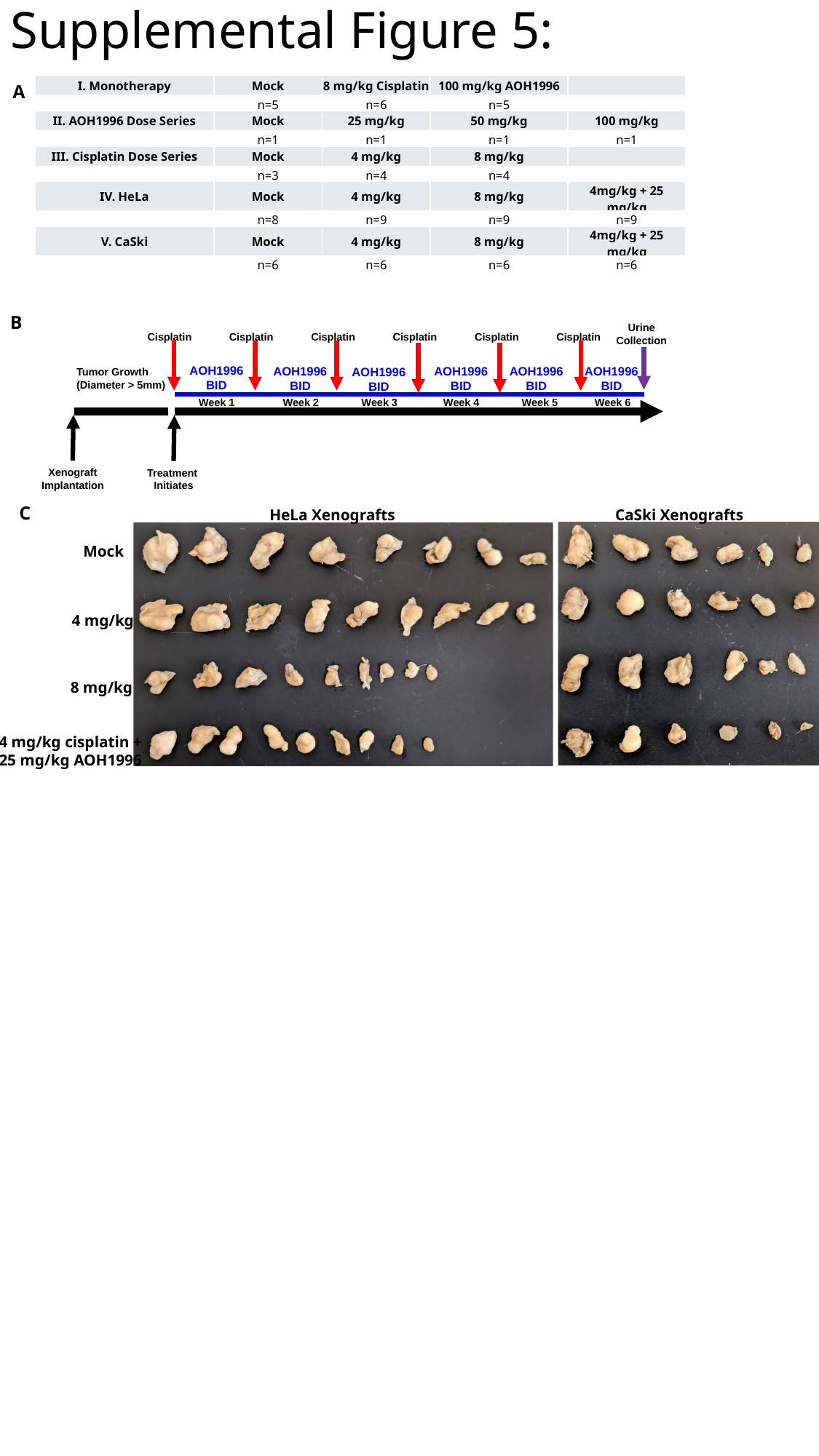

Supplemental Figure 5:
A
| I. Monotherapy | Mock | 8 mg/kg Cisplatin | 100 mg/kg AOH1996 | |
| --- | --- | --- | --- | --- |
| | n=5 | n=6 | n=5 | |
| II. AOH1996 Dose Series | Mock | 25 mg/kg | 50 mg/kg | 100 mg/kg |
| | n=1 | n=1 | n=1 | n=1 |
| III. Cisplatin Dose Series | Mock | 4 mg/kg | 8 mg/kg | |
| | n=3 | n=4 | n=4 | |
| IV. HeLa | Mock | 4 mg/kg | 8 mg/kg | 4mg/kg + 25 mg/kg |
| | n=8 | n=9 | n=9 | n=9 |
| V. CaSki | Mock | 4 mg/kg | 8 mg/kg | 4mg/kg + 25 mg/kg |
| | n=6 | n=6 | n=6 | n=6 |
B
Urine Collection
Cisplatin
Cisplatin
Cisplatin
Cisplatin
Cisplatin
Cisplatin
AOH1996 BID
AOH1996 BID
AOH1996 BID
AOH1996 BID
AOH1996 BID
AOH1996 BID
Tumor Growth (Diameter > 5mm)
Week 1
Week 2
Week 3
Week 4
Week 5
Week 6
Xenograft
Implantation
Treatment
Initiates
C
HeLa Xenografts
CaSki Xenografts
Mock
4 mg/kg
8 mg/kg
4 mg/kg cisplatin +
25 mg/kg AOH1996

## Slide 2
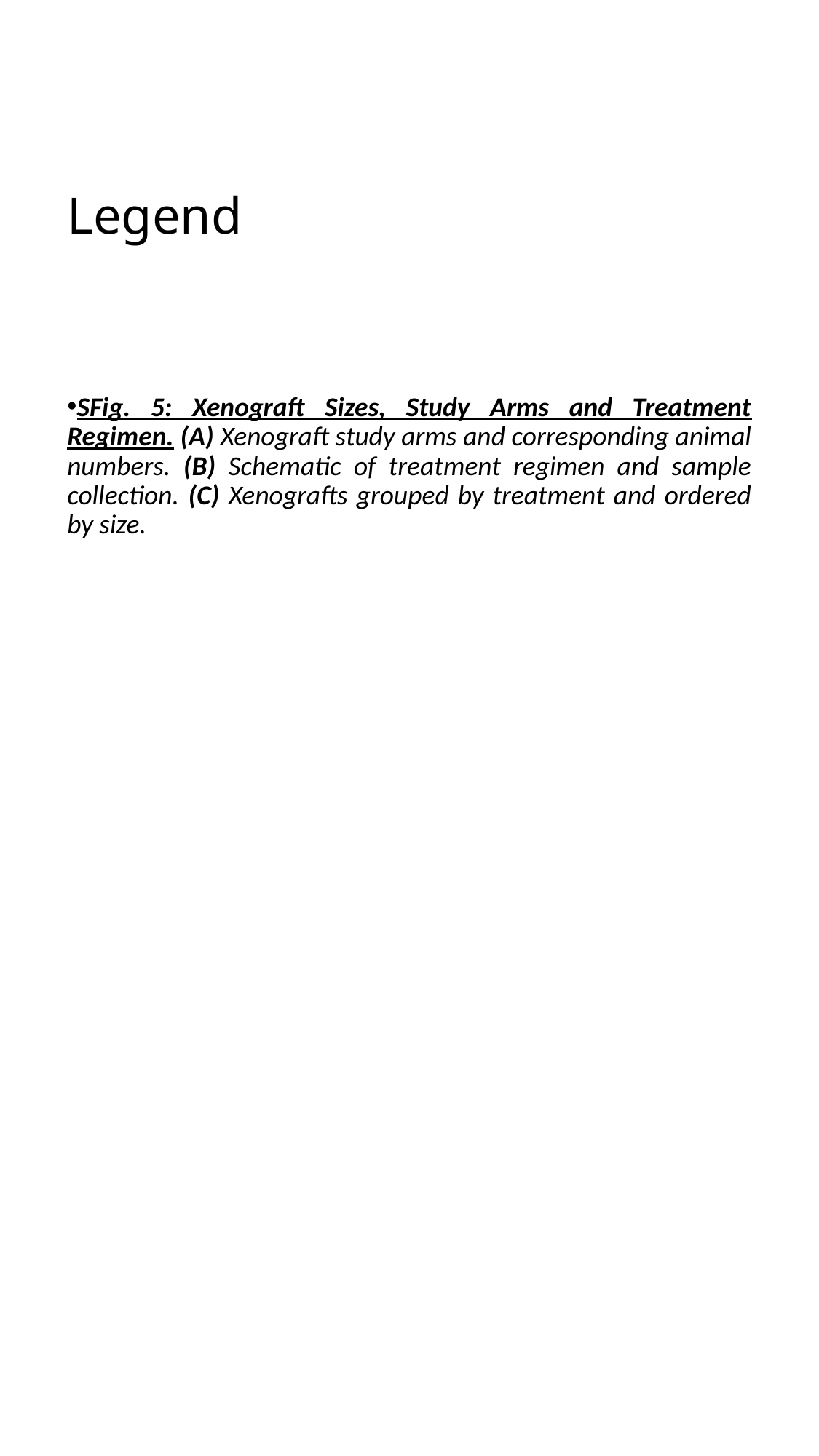

# Legend
SFig. 5: Xenograft Sizes, Study Arms and Treatment Regimen. (A) Xenograft study arms and corresponding animal numbers. (B) Schematic of treatment regimen and sample collection. (C) Xenografts grouped by treatment and ordered by size.
