# Supplementary material for: Targeting Cancer-Associated PCNA with AOH1996 Induces Mitotic Catastrophe and Enhances Cisplatin Therapy in Cervical Cancer
Source: Cancer Res Commun. 2026 May 27;6(5):1220–38. doi: 10.1158/2767-9764.CRC-25-0648 (PMC13213708; doi:10.1158/2767-9764.CRC-25-0648)
Supplement: Supplemental Figure 6 — H&E Analysis Did Not Detect Gross Deferential Nephrotoxicity. [file crc-25-0648_supplemental_figure_6_suppsf6.pptx]

## Slide 1
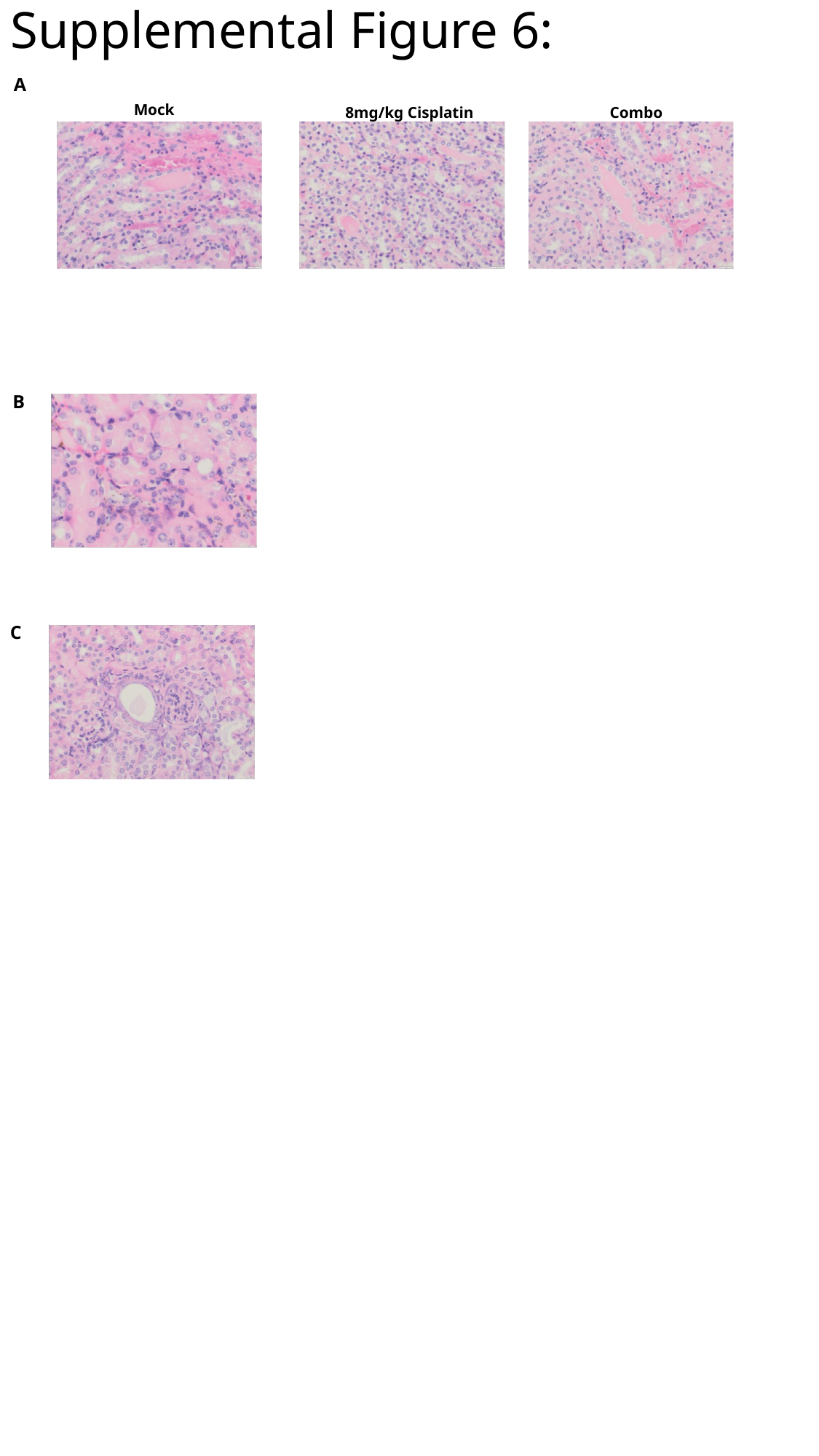

Supplemental Figure 6:
A
Mock
8mg/kg Cisplatin
Combo
B
C

## Slide 2
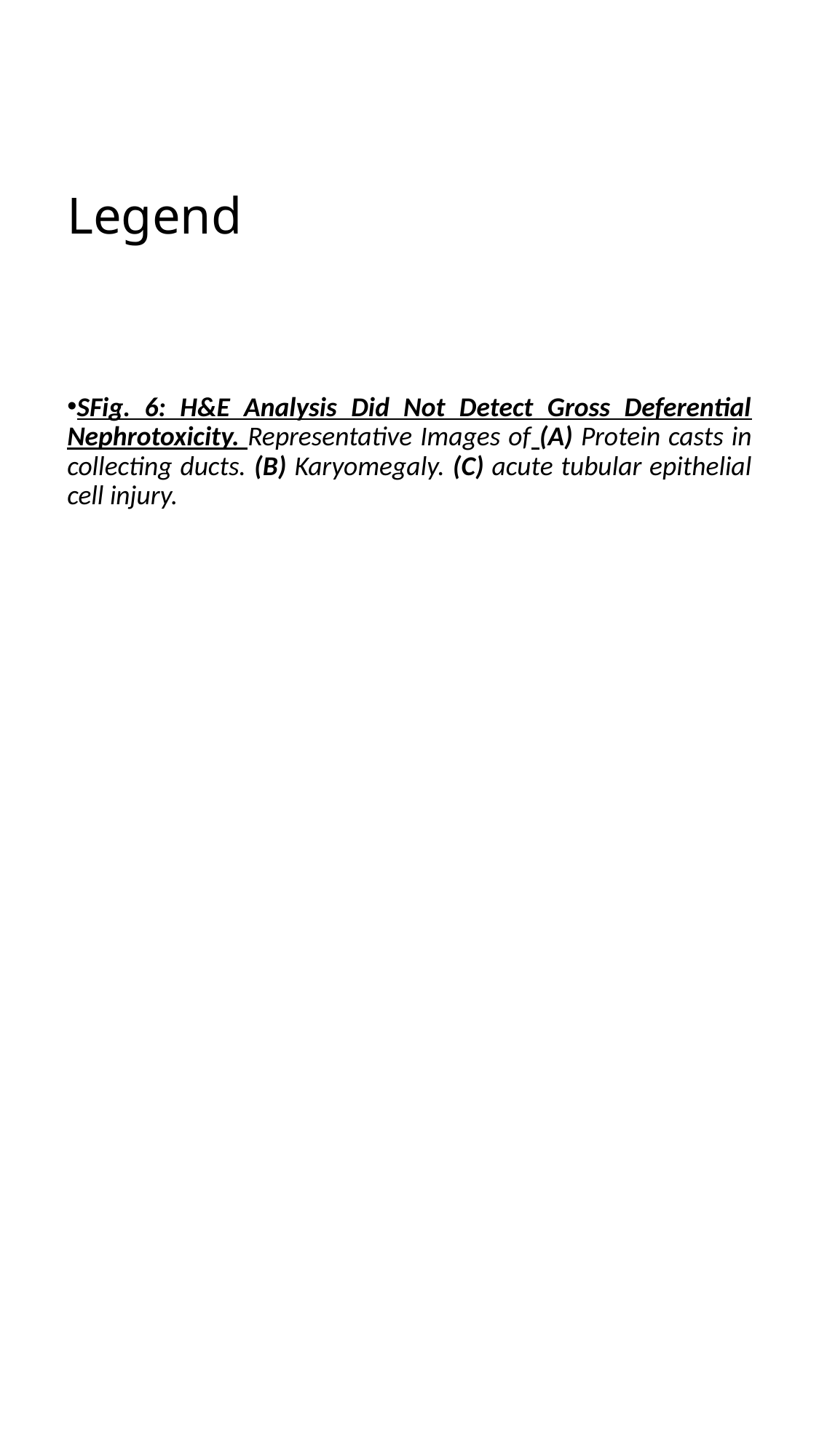

# Legend
SFig. 6: H&E Analysis Did Not Detect Gross Deferential Nephrotoxicity. Representative Images of (A) Protein casts in collecting ducts. (B) Karyomegaly. (C) acute tubular epithelial cell injury.
